# Characteristics of pre-metastatic niche: the landscape of molecular and cellular pathways

**DOI:** 10.1186/s43556-020-00022-z

**Published:** 2021-01-30

**Authors:** Hao Wang, Junjie Pan, Livnat Barsky, Jule Caroline Jacob, Yan Zheng, Chao Gao, Shun Wang, Wenwei Zhu, Haoting Sun, Lu Lu, Huliang Jia, Yue Zhao, Christiane Bruns, Razi Vago, Qiongzhu Dong, Lunxiu Qin

**Affiliations:** 1 Department of General Surgery, Huashan Hospital & Cancer Metastasis Institute & Institutes of Biomedical Sciences, Fudan University, 12 Urumqi Road (M), Shanghai, 200040 China; 2 Avram and Stella Goldstein-Goren, Department of Biotechnology Engineering, Ben-Gurion University of the Negev, Beer-Sheva, Israel; 3 Medical Faculty, University of Cologne, Cologne, Germany; 4 Department of General, Visceral, Cancer and Transplantation Surgery, University Hospital of Cologne, Cologne, Germany

**Keywords:** Pre-metastatic niche, Extracellular vesicles, Bone marrow-derived cells, Vascular alteration, Immunosuppression, Therapeutic strategies, Metastasis

## Abstract

Metastasis is a major contributor to cancer-associated deaths. It involves complex interactions between primary tumorigenic sites and future metastatic sites. Accumulation studies have revealed that tumour metastasis is not a disorderly spontaneous incident but the climax of a series of sequential and dynamic events including the development of a pre-metastatic niche (PMN) suitable for a subpopulation of tumour cells to colonize and develop into metastases. A deep understanding of the formation, characteristics and function of the PMN is required for developing new therapeutic strategies to treat tumour patients. It is rapidly becoming evident that therapies targeting PMN may be successful in averting tumour metastasis at an early stage. This review highlights the key components and main characteristics of the PMN and describes potential therapeutic strategies, providing a promising foundation for future studies.

## Introduction

Tumour metastasis accounts for a large proportion of cancer-associated mortalities. Despite the progress that has been made in this area, the complex-stepwise process of tumour cell dissemination to target organs is yet poorly understood [1]. However, it is widely accepted that a pre-metastatic niche (PMN) offers an opportunity for primary tumour cells to efficiently adapt and survive, even within hostile microenvironments. As a result, tumour cells can multiply and enable the formation of subsequent metastasis [2]. The original concept of the PMN dates back to 1889 when Stephen Paget proposed the “seed and soil” hypothesis, that is, the tumour dissemination is governed by the crosstalk between tumour cells (the “seeds”) and host organ (the “soil”) [3]. A PMN is created by the primary tumour and can acquire permissive and supportive properties allowing colonization at target organs. The purpose of this review is to provide a perspective on our understanding of the characteristics and mechanisms involved in PMN establishment and progression.

## Underlying mechanisms in the induction and formation of the PMN

Numerous studies have established that various cellular components and signalling pathways are responsible for its formation in different tumour models. The initiation and formation of the PMN are regulated through different mechanisms, allowing disseminating tumour cells to colonize future metastatic sites.

### Extracellular vesicles

Extracellular vesicles (EVs), a collective term covering a variety of cell-derived membranous structures, can encapsulate and transport various cellular materials [4–8], mediating the crosstalk between primary tumour microenvironment and the PMN during the early stages of tumour metastasis [9, 10]. Tumour-derived EVs can alter the microenvironment in future metastatic sites by directly targeting organ-specific resident cells (liver Kupffer cells [11–14], hepatic stellate cells (hStCs) [12, 15], bone marrow stromal cells [16], tissue-resident macrophages [17, 18], lung fibroblasts [14, 15, 19–23], lung epithelial cells [14, 24], brain astrocytes [19], neurons [19] and microglia [25]). In this way, EVs facilitate PMN formation through induction of cytokines, chemokines and growth factors, extracellular matrix (ECM) remodelling and metabolic reprogramming. Apart from the pro-metastatic effects on these resident cells within specific pre-metastatic organs, EVs participate in the modulation of bone marrow-derived cells (BMDCs) and a variety of immune cells as well. EVs derived from highly metastatic melanoma cells are capable of reprogramming bone marrow progenitors toward a pro-vasculogenic and pro-metastatic phenotype through transferring MET oncoprotein [26]. Tumour-derived EVs can directly mediate cytotoxic immune cell dysfunction in the PMN, ultimately promoting tumour metastasis [27, 28]. The contribution of tumour-derived EVs to PMN formation may be exacerbated by anti-metastatic treatment like chemotherapy [29].

Vascular destabilization, caused by upregulation of vascular permeability and induction of angiogenesis, is one of many key steps during PMN establishment [2]. Therefore, the modulation of endothelial cell activity is a crucial aspect of the pro-metastatic effect of tumour-derived EVs. Recently, the importance of EVs in vascular destabilization has been highlighted. Tumour-derived EVs that contain proteins like epiregulin [30] and CEMIP [31] or RNA fragments such as mRNAs [32] and microRNAs (miR-105 [33], miR-25-3p [34], miR-181c [35]) directly or indirectly promote vascular permeability and angiogenesis in the PMN via the upregulation of proangiogenic factors and the modulation of tight junction proteins in endothelial cells. These aforementioned findings suggest that EVs from highly aggressive tumour cells lead to PMN formation through various mechanisms that depend on tumour type and future metastasis sites, whereas EVs released from non-metastatic tumour cells can potently inhibit metastasis. An example of such non-metastatic EVs is exosomes from non-metastatic melanoma cells, containing high levels of surface pigment epithelium-derived factor (PEDF). They stimulate an innate immune response, through the Nr4a1 transcription factor-driven expansion of Ly6C^low^ patrolling monocytes in the bone marrow, resulting in a clearance of cancer cells in the PMN. More specifically, cancer cell clearance is brought about by the recruitment of NK cells and tumour necrosis factor (TNF)-related apoptosis-inducing ligand (TRAIL)-dependent killing of melanoma cells by macrophages [36].

Alteration of the microenvironment of secondary organs by the secretion of EVs from non-tumour cells can be seen in various tissues. For example, carcinoma-associated fibroblast (CAF)-derived EVs possess a greater ECM remodelling ability than salivary adenoid cystic carcinoma (SACC)-derived EVs in inducing the formation of lung PMN and consequently increasing SACC lung metastasis [37]. Moreover, BMDC-derived EVs effectively potentiate the activation of hStCs to induce an immunosuppressive microenvironment in the liver for tumour metastasis [38]. EVs formed in metastatic organs may assist tumour cell proliferation and survival through the transmission of essential extrinsic signals to these cells [39]. Moreover, exosomal miR-23b delivered by bone marrow mesenchymal stem cells (MSCs) facilitates breast cancer cell dormancy within the PMN by suppressing target gene *MARCKS* [40]. Additional studies are needed to deepen our understanding of tighter links between EVs and the indispensable processes in PMN formation and even in consequently tumour cell extravasation and colonization at pre-metastatic sites.

### Cellular signalling pathways implicated in the PMN

The establishment of the PMN at metastatic sites is facilitated by complex reciprocal signalling pathways among tumour cells from primary sites, myeloid cells from bone marrow, and resident cells from target metastatic sites at early stages of metastasis. The recruitment of various cells including BMDCs in target organs is a crucial event during PMN formation [2]. The chemokine receptors can transduce intracellular signals by binding with their homologous ligands, which is crucial for pre-metastatic recruitment of specific cells and the subsequent tumour colonization. The importance of CCR2/CCL2 signalling has been well identified in the pre-metastatic recruitment of BMDCs (Gr1^+^ inflammatory monocytes [41], CD11b^+^Ly6C^hi^Ly6G^−^ monocytes [42], granulocytic myeloid-derived suppressor cells (MDSCs) [43] and monocytic MDSCs [44]). Moreover, CXCR2/CXCL1 signalling also participated in the recruitment of granulocytic MDSC in the PMN [45]. Additionally, CCR4/CCL17 or 22 and CXCR4/CXCL12 are vital chemokine signalling pathways for the recruitment of immune cells (regulatory T cells (Tregs) [46–48], neutrophils [49, 50] and dendritic cells (DCs) [48]) and participate in the metastatic colonization of tumour cells as well [16, 47, 48, 51–53]. In addition to chemokine signalling pathways, many other signalling pathways give rise to alterations in the cellular composition of future metastatic sites. STAT3 signalling in CD11b^+^ myeloid cells, for example, enable CD11b^+^ myeloid cells to mediate sustained survival and proliferation both of their own and of other stromal cells in the PMN [54, 55]. Moreover, receptor activator of NF-κB (RANK)/RANK ligand (RANKL) signalling [46] and prostaglandin E_2_ (PGE_2_) /EP3 signalling [48] in DCs lead to the expansion of Tregs and the recruitment of DCs, contributing to the formation of the PMN. Complement C5a/C5aR signalling induces the recruitment of MDSCs [56] and the proliferation but not the recruitment of alveolar macrophages [57] in the PMN, leading to an immunosuppressive environment favourable for tumour metastasis. In addition, fibronectin/VLA-4 signalling [51], periostin/integrin α_v_β_3_ signalling [58] and p38 signalling [59] have proven to be responsible for the pre-metastatic accumulation of BMDCs.

Numerous studies have documented that signalling pathways in endothelial cells such as STAT3 signalling [60], Notch1 signalling [61], calcineurin-NFAT signalling [62] and CCR2/CCL2 signalling [42, 63] modulate the expression of cytokines, chemokines and adhesion molecules, which are, at least in part, responsible for adhesion and invasion of tumour cells in the PMN. Moreover, STAT3 signalling [64] and mTOR signalling [65] in lymphatic endothelial cells (LECs) within the PMN facilitate tumour cell extravasation and colonization. Apart from endothelial cells, signalling pathways in other organ-specific non-myeloid cells also have a role in the establishment of the PMN. In pre-metastatic bone, RANK/RANKL signalling creates an osteolytic PMN through the facilitation of pre-osteoclast maturation, which induces the release of soluble and insoluble factors from the skeleton, supporting cancer growth and survival in bone [66, 67]. Shh signalling [67] and Notch signalling [68] in osteoblasts confer proliferative advantages to bone metastatic tumour cells through the production of interleukin (IL)-6. In the pre-metastatic liver, STAT3 signalling in hepatocytes induces the expression of serum amyloid A (SAA) 1 and SAA2 that are crucial for PMN formation [69]. The fact that STAT3 signalling induces the secretion of factors responsible for the formation of the PMN has also been demonstrated in tumour cells [54] and tumour-associated macrophages (TAMs) [70] within the primary tumour microenvironment. Tissue inhibitor of metalloproteinases (TIMP)-1/CD63 signalling in hStCs induces the expression of stromal-derived factor 1 (SDF-1, also known as CXCL12), which facilitates neutrophil recruitment for PMN formation [50]. Additionally, NF-κB signalling was reported to induce an inflammatory state in PMN to promote metastatic progression [15, 17, 23, 71]. A precise understanding of the contribution of signalling pathways to the dynamic process of PMN formation is a prerequisite to better understand the complex mechanisms involved and to design better therapeutic approaches targeting these signalling pathways to prevent tumour metastasis.

### Hypoxic control of the PMN

Hypoxia and activation of hypoxia-inducible factor (HIF) signalling influence multiple steps within the tumour metastatic cascade [72]. Direct evidence for the role of hypoxia in the promotion of PMN formation is demonstrated by the lysyl oxidase (LOX) family proteins. During the early stage of tumour metastasis in breast cancer [73–75], colorectal cancer [76] and hepatocellular carcinoma [77], hypoxia acting as an important primary tumour microenvironmental factor regulates PMN formation through inducing the several members of LOX family, including LOX, LOX-like (LOXL) 2 and LOXL4. They can promote osteoclastogenesis in a RANKL-dependent or -independent manner and can modify ECM through catalysing collagen cross-linking in the PMN. By this, they are facilitating the recruitment of BMDCs and the following colonization of circulating tumour cells (CTCs). These findings demonstrate LOX family proteins as important biomarkers to identify pathways of PMN formation induced by hypoxia.

Importantly, the contribution of hypoxia to the formation of the PMN is not only through the LOX family proteins. Primary tumour hypoxia, for example, provides cytokines and growth factors capable of establishing a PMN through the recruitment of CD11b^+^Ly6C^med^Ly6G^+^ myeloid cells (driven partly by hypoxic tumour cell-derived monocyte chemotactic protein-1 (MCP-1), also known as CCL2) and a reduction in the cytotoxic effector functions of NK cell populations (attributed to the concomitant increase of CD11b^+^Ly6C^med^Ly6G^+^ myeloid cells) [43]. Moreover, HIF-1-induced carbonic anhydrase 9 (CA9) expression is a requisite for the mobilization of granulocytic MDSCs, driven by the granulocyte-colony stimulating factor (G-CSF), to the breast cancer lung PMN [78]. In addition to accurately regulating the secretion of cytokines and growth factors, hypoxia also is capable of augmenting EV secretion from breast cancer cells through HIF-dependent expression of small GTPase RAB22A [79], which may be associated with PMN formation. Moreover, the secretion of exosomes by prostate cancer cells under hypoxia can facilitate ECM remodelling and BMDC recruitment in target organs [80]. Further studies focused on the exact role of hypoxia and HIF-dependent signalling in PMN are required to find several potential biomarkers and therapeutic targets that could be valuable for the detection and treatment of metastatic disease.

## Components involved in the PMN

The illusive PMN is established by cooperative efforts of various players that eventually create an enabling microenvironment at which metastatic cells can anchor and survive under the proper spatial and temporal cues. The formation of this organ-specific niche takes place following ignition by the long-distance transmission of factors from the primary tumour, rearrangement of the ECM components at the distant site and assembly of cells to and within the recipient tissue. Upon development of the primary tumour and assembly of its microenvironment, a series of causative signals are released by the tumour cells to facilitate the complex steps towards the colonization of remote tissues [2, 59, 81].

### Cellular components

The cellular assembly and involvement in the formation and development of the PMN are both complex and elusive. Furthermore, the spatiotemporal nature of these components is considered to vary between organs, tissues and may also be fine-tuned by specific niches [81, 82]. As a result, various pro-tumorigenic bone marrow-derived sentinel cells blaze the way for the development of the PMN.

Following the progression of the primary tumour and establishment of a flux of soluble factors such as transforming growth factor-β (TGF-β), VEGF-A, S100 and SDF-1, a variety of cells involve pre-metastatic recruitment. Kaplan et al. [51, 83] demonstrated that bone marrow-derived hematopoietic progenitor cells (HPCs) that express VEGF receptor (VEGFR) 1 are recruited at early stages of the metastatic development. Their arrival contributes to the establishment of focal permissive PMN in the lung before tumour invasion. These cells also co-express CD34, CD11b, c-kit, and Sca-1, which may support their stemness properties, along with their locomotion to defined PMNs within the target tissue. These cells also express integrin adhesion molecules, such as VLA-4 (α_4_β_1_), that support their localization in specific areas. Kaplan et al. [83] also suggested that accumulation and activation of the PMN is an evolving process that is further fuelled by these cells, for example by the production of matrix metalloproteinases (MMP) 9 that may degrade the basement membrane, accelerating the extravasation of more VEGFR1^+^ cells into the niche. There is evidence that immature myeloid cells are also recruited to the PMN at early stages [84]. Some recent studies suggest that neutrophils are pivotal contributors for early development of the PMN. The role of neutrophils in the regulation of PMN formation has been thoroughly reviewed by Jablonska et al. [85].

Once the early settlers are embedded within the microenvironment, they further contribute to the local microenvironment remodelling and the activation of residential cells and BMDCs. Fibroblasts are indicated to be a major cellular component of the primary tumour microenvironment and also a pivotal member of the PMN. Under normal regulation, fibroblasts are classified as mesenchymal cells harnessed in the ECM. In cancer progression, they can be activated and have been termed as CAFs. Upon activation, local fibroblasts are triggered to produce various signalling molecules such as SDF-1, TGF-β, S100A4, IL-6, CCL2, fibronectin and MMPs, which acting as ECM modifiers can attract CXCR expressing cells and forge proangiogenic and antiapoptotic permissive microenvironment within PMN [51, 86–88]. It is important to note, however, that CAFs are composed of multiple subpopulations that come from various origins, including reprogrammed resident tissue fibroblasts, bone marrow-derived MSCs, pericytes, adipocytes, and endothelial cells. So that the expression of various commonly used fibroblast markers is extremely heterogeneous and varies strongly between different CAF subpopulations [89, 90].

#### Pericytes

Pericytes are a group of cells that can be identified within the areas of vasculatures, embedded in the basement membrane and closely associated with the vessel lining cells. In contrast to what was commonly thought, they do not serve solely as structural milieu, but, via adhesion belt and gap junctions directly interact with endothelial cells and also provide the source for paracrine signalling pathways [91, 92]. Under normal homeostasis, pericyte encrustation is crucial for vessel remodelling, maturation, and stabilization. They also participate in the regulation of blood flow and vessel permeability. It is therefore understood that under normal conditions there is a tight regulation to maintain the balance between proangiogenic and antiangiogenic ques. [93]. In addition to their role during normal development, they contribute directly or indirectly to tumour growth, metastatic spread, and resistance to therapy [94, 95]. Various tumorigenic effectors such as oxygen tension, acidic pressure, expression of VEGF (of which, mostly VEGF-A)/VEGFR, platelet-derived growth factor receptor (PDGFR) and others propel the pericytes towards the proangiogenic condition. Under these conditions, the pericytes increase their coverage to sustain the fabrication of flimsy interwoven vasculature that supports the growth of tumour mass and also contributes to the resistance to antiangiogenic therapies. This is the reason why pericytes are strategically targeted by antiangiogenic agents. However, some experimental evidence reveals the failure in the clinical trials attempting to block pericytes [96]. Taking into account the paradigm of pericytes biology, their role in the metastatic progression is not yet fully understood.

The entity of pericytes has evolved from being a mesodermal and ectodermal origin cell type, which seems only to function in mechanical support, to acting as endothelial cells that maintain vasculature stabilization and to being an active player that closely interacts to maintain endothelial cells. They release signals to the surrounding and play a pivotal role in the fabrication designated to perivascular niches. Strong evidence identifies pericytes as stem cells capable of forming several other cell types with angiogenic, myogenic, chondrogenic and osteogenic potential [97]. The discovery of multipotent capacities of perivascular populations led to the concept of the existence of a vessel-mural niche [98]. However, due to the absence of a unique marker, tracking pericyte lineage has traditionally proven to be difficult [98, 99]. Yamazaki et al. [100] demonstrated that a subpopulation within the embryonic skin tissue unexpectedly derives from endothelial- and hematopoietic-derived cell lines. These experiments suggest that during development the origin of tissue pericytes is heterogeneous. It is therefore suggested that even pericytes that originate from the same tissue, may be heterogeneous in their origin [100]. Herein we submit that the role of pericytes during the development of the PMN may also be heterogeneous and tissue-dependent. Additionally, more experimental work should be carried out in order to understand their involvement in the metastatic progression.

#### Hematopoietic stem cells (HSCs)

The differentiation of hematopoietic stem cells (HSCs) into mature cells is tightly controlled by the microenvironment of bone marrow, in which different stromal cells and ECM components are working in concert to regulate HSCs activity, including their mobilization, differentiation and quiescence. There are two distinct niches in the bone: the endosteal niche lining the bone surface and the perivascular niche around sinusoids. With regard to the perivascular niche, a variety of processes are working together to regulate HSCs. Some cell-regulating pathways, such as, Akt and p42/44 MAPK, can promote HSC expansion and self-renewal by upregulating IGFBP2, fibroblast growth factor (FGF) 2, BMP4 and DHH, and downregulating dickkopf (DKK1), which is an inhibitory factor of Wnt axis that controls HSCs self-renewal [99, 101, 102]. A variety of cell-cell and cell-ECM adhesion molecules such as E-selectin, E-cadherin and CD44 also take part in the regulation of HSCs within the perivascular niche. In cases of bone dwelling metastases, invading cells can take over both niches because like normal HSCs they can engage with the chemical, topological and molecular signalling of these niches [95]. It is now generally accepted that tumour cells are affected by oxygen tension, calcium flux and homing signalling such as SDF-1/CXCR4 and adhesion molecules, all of which mediate shuttling of cancer cells between the niches and between dormant and active states. However, the intricate influence of different bone marrow microenvironments on tumour development is currently under investigations.

#### MSCs

MSCs are the non-haematopoietic and multipotent cells with the capacity to differentiate into mesodermal lineages such as osteocytes, adipocytes and chondrocytes as well as ectodermal (neurocytes) and endodermal lineages (hepatocytes) [103]. Human MSCs were reported in bone marrow for the first time [104] and are continuously attracting attention because of their biotechnological and clinical potential [105]. Over a decade ago, MSCs was observed to be derived from a variety of tissues such as fat, skin, heart, muscle and liver. Interestingly, cells with MSC markers also express pericyte markers, so it was then suggested that all MSCs are pericytes [106] or derived from pericytes [107]. However, the progenitor hierarchy between MSCs and pericytes was recently challenged by different lineage tracing strategies. Guimarães-Camboa and colleagues showed that pericytes and vascular smooth muscle cells, although multipotent in vitro, do not behave as MSCs in vivo [94]. These findings contrast with previous studies that also used lineage tracing assays and give rise to an ongoing debate regarding the progenitor hierarchy between MSCs and pericytes [94, 108]. Both MSCs and pericytes take an active role in the formation of the PMN. To some extent, it may be suggested that this MSCs/pericytes tangle is a causative factor that regulates extravasation, adhesion and dormancy-reactivation.

It is well documented that MSCs closely interact with the tumour microenvironment and affect tumour progression. However, it should be considered that experimental publications may reflect contradicting results on the role of MSCs as tumour promotors or inhibitors and the molecular and cellular mechanisms of their interactions are poorly understood [109]. Several studies indicate that MSCs are significant players in the formation of distant metastatic sites. For example, Karnoub and colleagues [110] showed that CCL5 secreted by MSCs promotes lung metastasis of breast cancer through binding its receptor CCR5. It was also found that tumour-derived osteopontin induces the production of CCL5 by the MSCs [111]. As a part of the homing cascade, SDF-1 from MSCs and their terminal differentiation, osteoblasts, interacts with its receptor CXCR4, which is highly expressed by bone metastasizing lineages like breast and prostate, facilitating tumour cells to colonize and survive in the bone microenvironment [105, 112, 113].

It is now widely accepted that just a small subset of invading tumour cells can survive the mechanical, physical and immunological pressures and harness the new terrain. This subpopulation of cells is characterized by dedifferentiation and stem-like traits relating to the properties of metastasis initiation [114]. The metastatic capabilities of these cancer stem cells (CSCs) are remarkable since they are able to evade and survive the immune response, resist therapeutic agents, enter dormancy and reactivation. All of these stem-like properties create a well-adjusted “spearhead” that can penetrate, integrate and, later, further modulate the permissive PMN [115, 116]. The interaction between tumour cells and their niches including MSCs is critical for supporting CSC preservation and later development [117, 118].

### ECM components

The ECM is a complex bio-lattice whose composition and structural characteristics vary among different tissues to support their specific functional needs. In many cases, the ECM is a highly organized 3D woven structure with a pivotal role in shaping the structural, biochemical and molecular landscape of many tissues. The ECM with its morphological architecture provides the physical properties for cell anchorage to direct and support cell motility during embryonic development, stem cell migration and repair of diseased tissue. In addition to connective tissues, the ECM is highly expressed in the basement membrane of endothelial cells and vasculature. The ECM consists of numerous molecules that, for a long time, have been considered to be mainly mechanical constituents and scaffolding for cellular anchorage and architectural integrity. However, in the last two decades it became apparent that rather than serving simply as a mechanical intercellular entity, the ECM is an active milieu affecting a wide array of intercellular and intracellular processes. These are being executed mainly by fibronectin and collagens [119, 120]. ECM constituents take part in cell-cell communication, adhesion, and various signalling cascades controlling the fate of stem cells during embryogenesis and normal tissue homeostasis [121, 122]. The role of the ECM in the context of development and maintenance of the stem cell niche is of great importance and the action of the niche with its wealth of causative stem cell signalling mechanisms should be tightly regulated in a spatiotemporal manner [123]. A large body of evidence shows that the alteration of the stem cell niche plays a key role in a number of diseases associated with tissue degeneration, ageing, and neoplasia [124]. In diseased tissues, particularly tumours, the ECM undergoes intensive modifications along with interplay with the primary tumour that heavily influence tumour progression.

Yet, it is not always clear which occurs first, abnormal ECM formation that nurses tumour development or abnormal cellular and molecular processes that ultimately alter ECM structure and function in such a way that enables the occurrence of a tumour supportive crosstalk. Beyond these short-distance interactions, tumour-derived factors have been recently shown to depart the tumour microenvironment, circulate through the body and exert effects on ECM within distant organs [125]. These, along with other long-distance modifications, act in concert to establish the PMNs. In this permissive microenvironment, ECM molecules can interact with the invading cells in different manners, such as mechanical 3D scaffolding, anchorage via cell surface receptors and release of entrapped growth factors and chemokines, which, in return, further prepares the PMN for cancer cells arrival [126–129]. Studies confirm that similarly to the primary tumour niche, the ECM and its components are essential players in tumour interactions and progression within the PMN. It is suggested that once they surpass a certain threshold, deregulated ECM dynamics may cause irreversible changes to the main ECM constitutes of a normal niche and convert it into a cancer-permissive microenvironment.

#### Collagens

Collagens, such as I, III, IV, V are primary ECM proteins. Their primary function is providing structural support and binding other ECM proteins. Collagens represent as much as 30% of the total mammalian protein mass [130]. Fibrillar collagens form fibrous structures mostly found in tendons, cartilage, skin and cornea. Each collagen fibre is made up of several subtypes of collagen as dictated by the particular tissue and function. Type I is the most abundant fibrillar collagen, found in connective tissues, such as skin, tendon, cornea and bone [130]. Type IV collagen is a key component of the basement membrane, which is found at the basal surface of epithelial and endothelial cells and is essential for tissue integrity [130]. Collagen I is heavily involved in processes such as wound repair and organ development [131]. As such, collagens take part in desmoplasia at metastatic sites where they may contribute towards the successful establishment of metastases. It is demonstrated that the architecture of the collagen scaffolds in tumours is severely altered. They are often linearized and crosslinked, reflecting elevated deposition and significant posttranslational modification [130, 132]. These collagen modifications further affect cellular metabolism, proliferation, differentiation, and apoptosis via signalling pathways, such as TGF-β/Smad. The crosstalk between collagen and cancer cells is executed mainly by direct anchorage to the cancer cell receptors. Adhesion of collagen I and collagen IV to cancer cells impacts cancer progression. The cadherin family represents one typical cell adhesion molecule that is closely related to collagen activity. Collagen I stimulates E-cadherin upregulation to facilitate the migration of pancreatic ductal adenocarcinoma (PDAC) cells [126, 133]. It is therefore suggested that these modified assemblies are involved in priming the construction of the PMN.

#### Fibronectin

It is well documented that fibronectin genes are upregulated in tumour cells during the epithelial-mesenchymal transition (EMT). Fibronectin is also considered, in some cases, as a biomarker for more aggressive mesenchymal characteristics. However, the role of fibronectin in tumorigenesis and malignant progression has been highly controversial and conflicting data is spanning from a tumour-suppressive to a pro-metastatic role associated with poor prognosis. Interestingly, fibronectin matrix deposited in the tumour microenvironment promotes tumour progression but, paradoxically, is also related to a better prognosis. Tsung-Cheng et al. [134] also suggested that the expression of fibronectin indicates acquisition of stemness state and drug resistance under which tumour cells grow significantly slower. They proposed that fibronectin is involved in the suppression of early tumour growth and progression but promotes late cancer metastasis, which may explain some of the contradictory studies. Libring et al. [135] recently demonstrated a dynamic relationship between the tumour and stromal cells within the tumour microenvironment in which the levels and fibrillization of fibronectin in the ECM are modulated during the particular stages of metastatic progression. Fibronectin has been shown to play a central role in processes associated with tumour progression. In particular, integrin α_5_β_1_ and fibronectin have not only been shown to be upregulated in tumours but have also been reported to participate in tumour cell proliferation [136]. Fogelgren et al. [137] suggested that the fibronectin matrix may provide a specific microenvironment to regulate LOX catalytic activity and Erler et al. [74] demonstrated that in the case of lung metastasis, after secreted by primary breast tumours, LOX co-localized with fibronectin within the domain of the PMN where it catalyses crosslink collagen IV in the lung basement membrane.

#### Hyaluronan (HA)

Hyaluronan (HA), is a large, high-molecular-weight, linear GAG composed of 2000 to 25,000 disaccharide units of glucuronic acid and N-acetylglucosamine [138]. It is now well documented that HA is not just a space filler but a biomolecule having multifunctional capacities. HA is highly expressed during early development and regulates essential biological processes by mediating cell activities such as migration, proliferation and differentiation. In addition to its ability to expand its volume by many folds, other interactions and functions are being executed via an array of cell surface receptors, including the receptor for hyaluronan-mediated motility (RHAMM), Toll-like receptor (TLR) 4 and CD44, the prominent HA receptor [139]. CD44 isoforms are overexpressed in several cell types including CSCs, hence considered to have a role in cancer progression [140, 141]. Accumulating evidence indicates that CD44 isoforms, especially CD44v isoforms, are CSC markers and critical players in regulating the properties of CSCs, including self-renewal, tumour initiation, metastasis and chemoresistance. In addition, it is becoming evident that CD44 is a signalling hub that integrates physical stimuli with growth factor and cytokine signals and transduces signals to membrane-associated cytoskeletal proteins or to the nucleus. This allows regulation of a variety of gene expression signals affecting cell-matrix adhesion, cell migration, proliferation, differentiation, and survival [141]. Since CD44 binds to several ligands such as HA, osteopontin, chondroitin, collagen and fibronectin that can be modulated during metastatic progression, it represents an important affector of the PMN. In addition, during assembly of the PMN, CD44 is required for the CD44v6-mediated assembly of a soluble matrix that supports exosomes for activating target cells in the PMN [142]. McFarlane et al. [143] reported that CD44 increases the efficiency of distant metastasis of breast cancer. They demonstrated that the loss of CD44 attenuated tumour cell adhesion to endothelial cells and reduced cell invasion, but did not affect proliferation in vitro. Avigdor et al. [144] demonstrated crosstalk between CD44 and CXCR4 signalling. They found that HA is expressed in human bone marrow sinusoidal endothelium and endosteum, the regions where SDF-1 is also abundant. This suggests a key role of CD44 and HA in SDF-1-dependent transendothelial migration of HSCs/HPCs and their final anchorage within specific niches of the bone marrow. It is therefore suggested that HA-CD44 and their effectors, which are taking an important role in the fine-tuning of various niches, are pivotal players in the formation and regulation of the PMN. However, further investigations should be carried out in order to shed light upon these important, yet elusive issues.

## Characteristics of the PMN

**Fig. 1:**
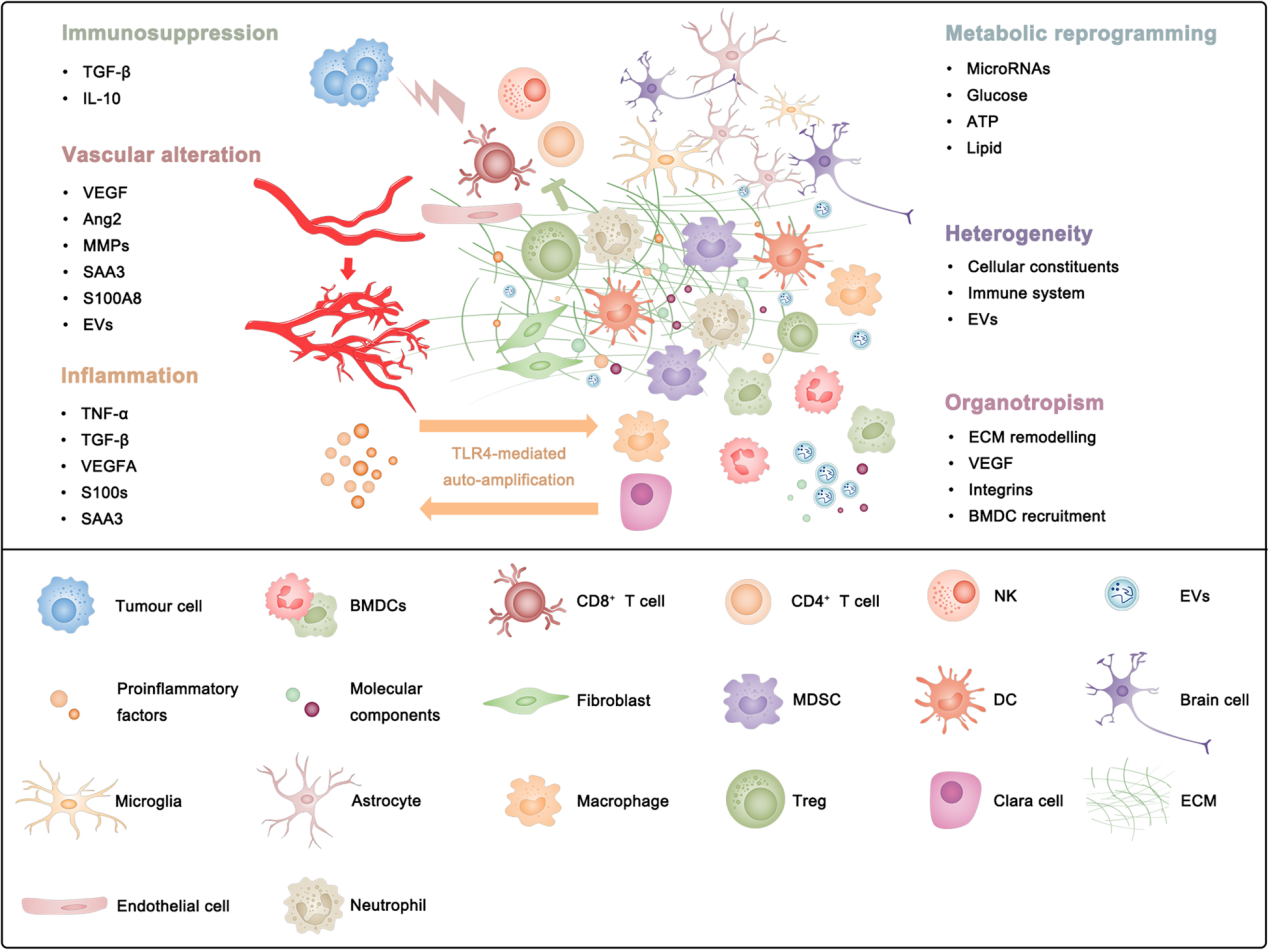
The main characteristics of the pre-metastatic niche (PMN) can be recognized as vascular alteration, immunosuppression, inflammation, metabolic reprogramming, heterogeneity and organotropism

PMN formation involves a series of dynamic changes in specific organs induced by tumour-derived factors and EVs during the early stages of tumour metastasis [9, 10, 145–148]. The secretion of various soluble factors and EVs, by primary tumours, allows mediation of the informational transfer from local to near or distant sites and seems to be the earliest event in the formation of the PMN. It is then followed by the modification of local microenvironment, including the functional altering of resident cells, the remodelling of ECM and the recruiting BMDCs and other types of immune cells [2, 127, 149–151]. During this dynamic process, special characteristics of the PMN have been recognized as critical to favour efficient tumour cell colonization and outgrowth of nascent metastases (Fig. 1).

### Vascular alteration

Accumulated studies have demonstrated that the vasculature at PMNs is remodelled to suitable for the extravasation of tumour cells. VEGF, Ang2 and MMPs are responsible for the disruption of vessel stability in the PMN. For example, pre-metastatic factors such as Ang2, MMP3 and MMP10, which are upregulated in the pre-metastatic lungs by primary tumours, can disrupt the vascular integrity synergistically to facilitate the extravasation of tumour cells [152]. In another preclinical experiment, Ang2 upregulation in the pre-metastatic lungs was identified as a result of calcineurin-NFAT signalling activation in lung endothelium, and as a link between increased VEGF in the lung and increased angiogenesis in the metastatic niche promoting lung metastases [62]. MMP9 provided by recruited Gr1^+^CD11b^+^ myeloid cells promotes decreased pericyte coverage and disruption of VE-cadherin junctions in vascular endothelium, contributing to aberrant and leaky vasculature in the pre-metastatic lung [153]. Additionally, miR-30s that modulate pre-metastatic lung vessels mainly through MMPs are downregulated in fibroblasts of pre-metastatic lungs, leading to vascular destabilization and subsequent extravasation and colonization of tumour cells [154].

The dysregulation of tight junctions is another crucial factor for the deconstruction of the integrity of endothelial barriers in the PMN. Breast cancer cell-derived exosomes containing miR-105, for example, increase metastasis at PMNs by destroying the endothelial cell barrier through downregulating the tight junction zonula occludens 1 (ZO-1) protein [33]. Exosomal miR-25-3p derived from colorectal cancer cells regulates the expression of VEGFR2, ZO-1, occludin and Claudin5 in endothelial cells by targeting KLF2 and KLF4, consequently promoting vascular alteration in the PMN [34]. Inflammatory factors such as SAA3 and S100A8 were reported to trigger the formation of regions of hyperpermeability via TLR4 and its coreceptor MD-2 in the pre-metastatic lungs, increasing pulmonary susceptibility to metastatic homing [63]. Moreover, uptake of CEMIP^+^ tumour exosomes by brain endothelial and microglial cells induces endothelial cell branching and inflammation in the perivascular niche by upregulating the proinflammatory cytokines encoded by *Ptgs2*, *Tnf* and *Ccl/Cxcl*, known to promote brain vascular remodelling and metastasis [31]. In addition, a marked increase in lymphatic vessel density and a specific capsular and subcapsular distribution are observed in pre-metastatic sentinel lymph nodes of patients with early cervical cancer [155], which highlights the important role of lymphatic vascular remodelling in PMN. Exosomal IRF-2 induces the release of lymphangiogenic factors VEGF-C by macrophages, promoting sentinel lymph node metastasis of colorectal cancer [156]. The LECs within the lungs and lymph nodes are affected by tumour-secreted factors and start to express CCL5, which is neither expressed in normal LECs nor in cancer cells, and VEGF, which enhances lung vascular permeability and induces lymph node angiogenesis, promoting metastatic extravasation and colonization [64]. Furthermore, heparin-binding factor midkine, secreted by tumour cells, facilitates lymphangiogenesis through paracrine activation of the mTOR signalling pathway in LECs, thus promoting metastasis progression [65].

### Immunosuppression

As a significant barrier to metastasis, the immune system has a critical role in metastatic colonization in PMNs. Before their thriving in a future metastatic site, tumour cells must overcome immunological elimination by establishing an immunosuppressive PMN to protect them from induced apoptosis. Immunosuppression in the pre-metastatic lungs involves recruitment of MDSCs to these sites and regulation of immunosuppressive factors production, such as TGF-β and IL-10, which favours the generation of Tregs and polarization of CD4^+^ T cells to a Th2 type that renders CD8^+^ T cells dysfunctional [56, 58]. This data is consistent with a previous experimental result that Gr1^+^CD11b^+^ immature myeloid cells in pre-metastatic lungs decrease IFN-γ and elevate Th2 cytokine production [153]. In another experiment, recruited granulocytic MDSCs proved to be the main source of IL-10, which inhibits the activity of T cells in pre-metastatic lungs [157]. Furthermore, the reduction of cytotoxic effector functions of NK cells may also be attributed to the increase in granulocytic MDSCs in the pre-metastatic lungs [43]. Additionally, epithelial Notch1 signalling triggers CXCR2-dependent Ly6G^+^ neutrophil accumulation within the PMN and generates an immunosuppressive environment featured with decreased infiltrating CD8^+^ T cells [158]. In the pre-metastatic lymph nodes, DC-derived SDF-1 may be responsible for attracting Tregs to these sites, which influences the fate of metastasized tumour cells [48]. Moreover, recruited Tregs in bone marrow may form an immunosuppressive niche facilitating the bone metastasis [46]. Tissue-resident macrophages such as alveolar macrophages may also have important immunomodulatory functions in the PMN. CD11b^neg^F4/80^+^ alveolar macrophages preconditioned by breast tumours not only inhibit Th1 and favour generation of Th2 cells that had lower tumoricidal activity than Th1 cells but also reduce the number and maturation of lung DCs by regulating TGF-β in the lung environment [57]. Microglia, as the major innate immune cells in the brain, have an important role in modulating the local immune response during brain metastasis. Microglia are reprogrammed by breast cancer towards a pro-metastatic phenotype, which upregulates immunosuppressive cytokines in microglia that inhibit T cell proliferation [25]. In addition to promoting the pre-metastatic recruitment of immunosuppressive cells, primary tumour cells can directly mediate the dysfunction of anti-tumour effector cells such as T cells and NK cells via EVs, leading to the suppression of anticancer immune responses in pre-metastatic organs [27, 28]. Tumour cells can evade immune surveillance by the expression of programmed death-ligand 1 (PD-L1), which interacts with programmed death-1 (PD-1) receptor on T cells to elicit the immunosuppressive response [159]. Given that metastatic tumours can release EVs, mostly in the form of exosomes, that carry PD-L1 on their surface [160] and that PD-1 is upregulated in pre-metastatic sentinel lymph nodes [155], it is very likely that the immune dysfunction in the PMN might be induced by exosomal PD-L1 released by primary tumour cells. Altogether, suppression of local antitumor immunity and the contribution of regulatory immune cells in PMN ultimately result in immunosuppressive PMN formation to facilitate metastatic seeding.

### Inflammation

Inflammatory alterations in target metastatic sites may facilitate dynamic evolution of the PMN and the ensuing invasion of target organs by tumour cells. In recent years, a variety of S100 family proteins have been found to play the role of inflammatory mediators in chronic inflammatory diseases, as well as soluble factors involved in the interactions between tumour and stromal cells during the PMN formation [161, 162]. Proinflammatory S100A8 and S100A9, whose expression is induced by factors secreted from primary tumours such as TNF-α, TGF-β and VEGF-A, serve as recruiters of CD11b^+^ myeloid cells into the pre-metastatic lungs, in turn promoting metastasis formation [59]. SAA3, an important proinflammatory downstream mediator of S100 family proteins, has a role in the accumulation of CD11b^+^ myeloid cells and can induce its own secretion by a positive feedback mechanism that depends on TLR4, a functional receptor for SAA3 in endothelial cells, macrophages and Clara cells within the pre-metastatic lungs [71, 163]. It has been found that an antagonist of the TLR4/MD-2 complex can inhibit both the recruitment of MDSCs and the induction of S100A8 and SAA3 in pre-metastatic lungs [164]. Moreover, SAA3 can also induce expression of the inflammatory cytokine TNF-α in both alveolar type II cells and macrophages, and TNF-α, conversely, directly activates the SAA3 promoter in alveolar type II cells [163]. Another study has reported that tumour-released S100A7 could be involved in the PMN formation, increasing tissue fibrosis, which may favour the implantation of BMDCs and, in the end, the colonization of the target organ by CTCs [165]. In addition, S100A4, another S100 family member, seems to induce SAA expression and other transcriptional targets in an organ-specific manner. S100A4 significantly increases the expression of SAA1, SAA3, RANTES, G-CSF, S100A8 and S100A9 in the liver, while increasing the expression of SAA1 and S100A9 but downregulates G-CSF and has a negligible effect on SAA3, RANTES and S100A8 in the lung [166]. These findings suggest that the reciprocal interplay of S100 proteins, SAA proteins and TNF-α serves to produce a proinflammatory milieu in PMNs that recruits BMDCs and subsequently attracts tumour cells, promoting metastatic progression.

### Metabolic reprogramming

Dysregulated metabolism, one of the hallmarks in cancer, influences metastasis and has been shown to play a crucial role in PMN. It is well known that metastatic tumour cells require a specific pattern of energy, nutrient, and oxygen to compete with the resident niche cells, thus adapt to the microenvironments of local tissues to establish a metastatic colony. Tumour-derived EVs can modulate the metabolism of stromal cells within specific organs, contributing to the creation of a PMN that promotes the development of metastasis. Breast cancer-derived extracellular miR-122, for example, can suppress glucose metabolism of resident cells in the PMN through the downregulation of the glycolytic enzyme pyruvate kinase, thus facilitating metastasis [19]. Human melanoma-derived exosomes that contain miR-155 and miR-210 are capable of reprograming the metabolism of stromal fibroblasts to increase aerobic glycolysis and decrease oxidative phosphorylation, consequently causing extracellular acidification of microenvironments in distal regions accessible to exosomes [167]. In addition to stromal cells within the PMN, bone marrow neutrophils seem to undergo dynamic metabolic changes. A recent study showed that bone marrow granulocytic MDSC-like neutrophils from the early stage of tumour-bearing mice had high glucose uptake, increased oxidative phosphorylation, increased tricarboxylic acid cycle flux and increased glycolysis, which resulted in a substantially greater production of ATP than that of neutrophils from tumour-free mice [168]. Obesity suppresses the expression of NK cell effector molecules by inducing metabolic reprogramming in NK cells to lipid metabolism [169], which may associate with immunosurveillance dysfunction in the PMN under the condition of obesity. Tumour cells need to adapt the activity of their metabolic pathway to the nutrients in the PMN as nutrient availability may differ between organs. In the liver, for example, colorectal cancer cells, by secreting creatine kinase, convert creatine and ATP into phosphocreatine that is subsequently taken up to generate intracellular ATP, which sustains the energetic requirements of colon cancer cells encountering hepatic hypoxia, allowing them to survive this barrier to metastatic progression [170]. Furthermore, colon cancer cells can upregulate aldolase B, an enzyme involved in fructose metabolism, to meet their need for energy during metastatic proliferation in the liver [171]. Thrombopoietin, as a component of the physiological environment derived mainly from the liver, can promote metastasis of colorectal tumour-initiating cells to the pre-metastatic liver through the upregulation of lysine catabolism in these tumour cells to generate glutamate for liver colonization [172].

### Heterogeneity

It is becoming increasingly apparent that tumour cells are heterogeneous, which can cause differences in tumour growth, metastasis and drug sensitivity between patients, within the same patient and even within cells in the same tumour [173]. Similarly, PMNs are increasingly recognized as heterogeneous with specific subtypes of niche components governing the development or homeostasis of selective functions. Cellular constituents of the PMN may differ between experiments with the same type of primary tumour and the identical future metastatic organ. In some mouse models of breast cancer, pulmonary alveolar macrophages or TAMs contribute to the PMN in the lungs [57, 70]. However, in other studies, neutrophils are revealed to be the main immune cells recruited to the pre-metastatic lungs of mammary tumour-bearing mice, although these cells have a low frequency in the primary tumour microenvironment [174, 175]. This diversity implies that the formation of the PMN is a dynamic process and the difference in results may be due to the timing of the experiments. A recent study has shown that immune cells arrive at the pre-metastatic lung in waves differentially and sequentially, and some of the immune cells are only transiently present in the tumours [176]. In addition, the immune system of the PMN may be profoundly distinct. Neutrophils are regarded as critical regulators within the PMN, but they have a paradoxical role in the niche. In some animal models, neutrophils in the pre-metastatic lung inhibit the development of both breast and renal cancer lung metastases [174, 177]. In contrast, neutrophils are also demonstrated to be the main component and driver of metastatic establishment within the pre-metastatic lung microenvironment in several mouse breast cancer models [175, 178]. The paradoxical effects neutrophils possess may be contributed to the dynamic changes they undergo during the formation of the PMN, which is supported by a recent finding that bone marrow neutrophils in the early stages of lung cancer are functionally different from those in the late stages [168]. Tumour-derived exosomes are functional disparate in the establishment of the PMN. Currently, a growing body of evidence suggests that most metastatic cancers produce exosomes that condition PMNs in distant microenvironments to cause immunosuppression within the PMN [9]. However, other studies have revealed that pre-metastatic cancer exosomes trigger immune surveillance, which causes cancer cell clearance at the PMN by patrolling monocytes [36, 179]. Furthermore, it was shown that breast cancer cells with metastasis to lung or bone preferentially use OXPHOS over glycolysis, whereas liver-metastatic breast cancer cells incorporate glycolysis as the predominant metabolic process [180]. Therefore, the PMN is a highly complex, integrated ensemble of numerous components with unique functions and responds differently to the specialized tumour microenvironment.

### Organotropism

Different types of cancer present divergent tropisms to develop metastases in different organs. This organ tropism observed in metastasis, called “organotropism”, remains one of the most intriguing questions unanswered in cancer research. Recently, increasing numbers of studies attribute this organotropic metastasis pattern to the successful establishment of the PMN in specific organs, which educates and transforms the local milieu of the target organ into a microenvironment favourable for colonization and outgrowth of primary cancer cells. Organotropism in the establishment of the PMN may partly attribute to the different tendencies of ECM remodelling in various organs. For example, LOX has an important role of ECM remodelling in the pre-metastatic lungs while its expression and activity in other organs is reduced or remains unchanged, which is possibly explained by the high oxygen concentration in lung tissue that contributes to LOX enzymatic activity [181]. Moreover, fibronectin and periostin, the major ECM proteins, frequently deposit in the pre-metastatic lungs and increase metastasis by enhancing myeloid cell recruitment and through direct interactions with disseminated tumour cells (DTCs) [124]. Tumour-secreted growth factors and EVs may also dictate organotropic formation of the PMN. During primary Lewis lung carcinoma and B16-F10 melanoma growth, increased VEGF levels specifically in the lung, and no other organ microenvironments, trigger a threshold of calcineurin-NFAT signalling that transactivates *Ang2* in lung endothelium, thus inducing angiogenesis in the pre-metastatic lungs [62]. Additionally, tumour-derived exosomes can prepare the PMN in an organ-specific manner due to distinct integrin expression patterns, through which it was concluded that exosomal integrins α_6_β_4_ and α_6_β_1_ are associated with lung metastasis, while α_v_β_5_ with liver metastasis [14]. Furthermore, the site of BMDC recruitment is tumour type-specific. A previous study demonstrated that intradermal injection of Lewis lung carcinoma cells resulted in BMDC cluster formation limited to the lung and liver with no clusters in other organs, while the B16 melanoma tumour cells formed BMDC clusters in multiple tissues such as the lung, liver, testis, spleen and kidney, which are all common metastatic sites for this tumour [51]. In summary, primary tumour and stroma in future metastatic sites reciprocally influence each other and together determine the organotropism of the PMN.

## Significance of the PMN in cancer metastasis

The existence of the PMN has a significant role in cancer metastasis. The matured PMN is well-prepared for the seeding and growth of DTCs at metastatic sites. The stepwise progression of metastatic colonization and macrometastasis formation requires further fine-tuned crosstalk between the microenvironment and the metastatic cancer cells. The PMN can not only facilitate metastatic cell progression directly but can also induce their dormancy at metastatic sites for later recurrence.

### Dormancy and metastatic colonization

During metastatic colonization, cancer cells must shape themselves to better adapt to colonization and manipulate a favourable microenvironment at the metastatic site. Most of the DTCs undergo a state of dormancy when they arrive at a distant site, sustaining an equilibrium between growth and death. Dormancy is acquired through some pivotal traits of cancer cells and contributes to tumour recurrence years or even decades after primary tumour resection. DTCs prefer to remain in a dormant state instead of initiating outgrowth immediately, which helps DTCs resist cancer therapy, evading immune surveillance and survive for periods before entering a growth phase when colonizing [182, 183]. The fate of DTCs is a dynamic event that can be regulated by stromal cells, immune cells, ECM and a hypoxic microenvironment in PMN and can be controlled by intrinsic signalling such as endoplasmic reticulum stress, epigenetic and metabolism as well. A study demonstrated that breast tumour cell dormancy was regulated by perivascular niches in the lung, bone marrow and brain, in which endothelial-derived thrombospondin-1 induced sustained breast cancer cell quiescence. This suppressive cue was lost in sprouting neo-vasculature, which are characterized by reduced thrombospondin-1 expression and enhanced expression of pro-tumour factors periostin and TGF-β1 [184]. It has been found that differentiated osteoblasts can secrete soluble factors such as TGF-β2 and GDF10 that induce dormancy of prostate cancer cells via p38 MAPK activation in the bone metastatic niche [185]. DTCs of PDAC decreased the expression levels of major histocompatibility complex (MHC) class I, to help them evade immune surveillance [186]. In melanoma, CD8^+^ T cells have a key role in the maintenance of dormancy at the metastatic site, since deprivation of CD8^+^ T cells leads to a faster metastatic outgrowth [187]. Neutralization of IFN-γ, depletion of T cells or using PD-L1 blockade could reverse irradiation-induced dormancy in mouse models [188]. CXCL5 can promote the proliferation of breast cancer DTCs and its colonization in bone, in turn, blockade of CXCR2, the receptor of CXCL5, leads to DTCs dormancy [189]. During lung inflammation, two neutrophil extracellular trap (NET)-associated proteases, neutrophil elastase and MMP9, sequentially cleave laminin, which induces proliferation of dormant cancer cells by activating integrin α_3_β_1_ signalling [190]. Compared with primary colorectal tumours, collagen type I appears to be highly citrullinated in liver metastases, which leads to a decrease in migration and increase in adhesion of colorectal cancer cells and helps these cells to survive colonization [191].

In a perivascular niche, integrin-mediated interactions between DTCs with von Willebrand Factor (VWF) and vascular cell adhesion molecule-1 (VCAM-1) on the basal surface of bone marrow microvascular endothelium can protect DTCs from chemotherapy and promote bone metastasis [192]. Cell adhesion molecule L1 (L1CAM) activates Yes-associated protein (YAP) and myocardin-related transcription factor (MRTF) via engaging integrin β1 and integrin-linked kinase (ILK) in DTCs, which is a general requirement for the outgrowth of aggressive metastasis-initiating cells immediately after extravasation and of latent metastatic cells after exiting from quiescence [193, 194]. Versican produced by CD11b^+^Gr1^+^ myeloid progenitor cells in the pre-metastatic lungs stimulates mesenchymal-epithelial transition of metastatic tumour cells by attenuating phosphor-Smad2 levels, which results in elevated cell proliferation and accelerated metastases [195]. The research found that the activity ratio of ERK1/2 (extracellular signal regulated kinases) and p38 MAPK could switch on/off the dormancy of DTCs in human head and neck carcinoma. High expression of integrin α_5_β_1_, urokinase-plasminogen activator receptor (uPAR) and fibronectin induced activation of focal adhesion kinase (FAK) and Src kinase, thereby leading to high ERK/p38 ratio and the proliferation of dormant DTCs. In contrast, p38 MAPK activation may lead DTCs into a dormant state [196–199].

Recent studies prove that epigenetic alterations and non-coding RNAs (ncRNAs) regulate the cycles of dormancy and outgrowth of DTCs by mediating the activation of dormancy-associated master regulators. The orphan nuclear receptor (NR2F1) is a critical molecular regulator in dormancy maintenance of DTCs. NR2F1 could induce chromatin repression and quiescence of DCTs dependent on regulation of SOX9 and RARβ. Blockade of NR2F1 can interrupt DTCs dormancy and leads to tumour growth in metastatic sites [199]. In breast cancer cells, mitogen- and stress-activated kinase 1 (MSK1), a downstream effector of p38 MAPK, mediates the expression of key differentiation genes, including FOXA1 and GATA3 via easing the acetylation on Lysine 9 and 27 of histone 3, thereby facilitating DTCs proliferation and metastatic colonization [200]. The KDM family molecules, such as KDM2, KDM3B, KDM5B, KDM6 and KDM7, and histone deacetylases (HDACs) govern the dormancy or proliferation of DTCs through altered methylation and acetylation of histone 3 lysine residues, which affects the activation of key regulatory signalling and dormant DTCs reactivation [201–208]. In metastatic cells, PTEN-targeting microRNAs, derived from astrocytes in brain PMN, silenced its expression and increased the secretion of CCL2 chemokine, which led to IBA1-expressing myeloid cells recruitment and consequently led to the outgrowth of brain DTCs [39]. Metabolism alteration and hypoxic stress also have a critical role in regulating DTCs’ dormancy and metastatic colonization. Emerging evidence proves that activation of ferroptosis by glutathione peroxidase 4 (GPX4) may help cancer cells resist therapy and survive as “persister” cells. Co-treatment with kinase inhibitors and ferroptosis activators can eradicate drug-tolerant persister cancer cells [209, 210]. At distant metastatic sites, asparagine can increase the levels of HIF-1α and MYC when glutamine is scarce, which affects oxidant stress and EMT transition and induces DTCs to proliferate. If other nutrients are deficient in PMN, asparagine can promote glutamine biosynthesis and shape DTCs to survive and colonize in metastatic sites [211, 212].

### Remodelling the microenvironment for cancer metastasis

While DTCs arrive at the PMN from primary sites, they may encounter a hostile, anti-metastatic environment that induces apoptosis or necrosis. Thus, DTCs need to take an active role in inducing changes in the microenvironment to be able to survive and outgrow in the PMN. For initial survival, they directly or indirectly modulate the PMN by secreting factors that alter the composition of the niche and/or by instructing stromal cells in the niche to support the optimal metastasis-initiation. Tumours not only participate in the recruitment and expansion of immunosuppressive cell populations to create a permissive PMN but also modulate the function of immune cells towards a pro-metastatic phenotype to facilitate metastatic progression. Metastatic breast cancer cells, for example, can regulate the expression of inflammatory response genes in metastasis-associated macrophages (MAMs) in a VEGFR1-dependent manner, thus promoting metastatic growth [213]. Furthermore, a subset of VEGFR1^+^ MAMs that support angiogenesis and metastatic growth was identified in liver metastasis of colorectal cancer and found to be correlated with patient outcome [214]. MAMs expressing α_4_-integrins can promote breast cancer cell survival and metastatic growth via binding to the VCAM-1 on metastatic tumour cells [215]. Additionally, tissue-resident macrophages in omentum exhibit a functional diversification in the context of tumour growth and play a specific role in the malignant progression of DTCs and the development of invasive disease in a mouse model of metastatic ovarian cancer [216]. Similarly, neutrophils have been shown to be educated by metastatic microenvironment towards a metastatic-associated phenotype. In liver metastases of colorectal cancer, infiltrated metastasis-associated neutrophils expressing FGF2 promote the development of disorganized tumour vasculature and metastatic growth [217]. Moreover, it has also been found that neutrophils become more N2 polarized as breast cancer liver metastasis progresses and a greater fraction of neutrophils adopt an N2 phenotype when they are located close to the liver-metastatic lesions [218].

The crosstalk between metastatic tumour cells and stromal cells also plays an important role in the optimal modulation of metastatic progression. A study related to the cellular environment of metastatic breast cancer cells in the lung reported the presence of cancer-associated parenchymal cells, which exhibit stem cell-like features, express lung progenitor markers and exhibit multi-lineage differentiation potential and self-renewal activity [219]. In breast cancer, metastatic initiating cells mediate efficient lung fibroblast activation through thrombospondin-2 secretion, which is critical for efficient metastatic initiation within the lung tissue [220]. It has also been found that astrogliosis and neuroinflammation, physiologically instigated as a response of astrocytes to overcome brain tissue damage, is hijacked by brain-metastasizing tumour cells to support their growth [221]. In the liver metastatic niche, MAMs, by secreting granulin, activate hStCs into myofibroblasts that secrete periostin, consequently, turning the ECM into a fibrotic environment to support metastatic growth [222]. Moreover, breast cancer cells rely on the nutrient pyruvate to drive collagen-based remodelling of the ECM in the lung metastatic niche, supporting their own metastatic growth [223]. Lactate released by glycolytic breast carcinoma cells in the bone microenvironment is used as a fuel for the oxidative metabolism of osteoclasts, ultimately facilitating bone resorption without stimulating osteoclastogenesis to support metastatic growth [224]. Furthermore, the formation of blood and lymph vessels is conducive to environmental remodelling, which helps to provide an adequate supply for the initial tumour growth and subsequently metastasis. Accumulated studies demonstrated that the increased vascular permeability within the PMN is the initial step in the development of subsequent metastasis. In colorectal cancer, tumour-derived exosomal miR-25-3p in vascular endothelial cells promotes PMN formation, which dramatically induces vascular permeability as well as angiogenesis and enhances colorectal cancer metastasis in the liver and lung of mice [34]. In animal tumour models, the hyperpermeability of vessels within the PMN is associated with metastatic burden [152]. In pre-metastatic lungs, lung fibroblast-derived miR-30s stabilized pulmonary vessels and postponed metastasis. However, Skp2 directly targeted by miR-30s, could disrupt pulmonary vessels and promote lung metastasis [154]. Thus, the remodelling of the microenvironment by DTCs within a PMN in the secondary metastatic sites is competent to support the survival and outgrowth of DTCs, resulting in the pathological progression from micrometastases to significant macrometastases.

### Metastasis tropism

**Fig. 2:**
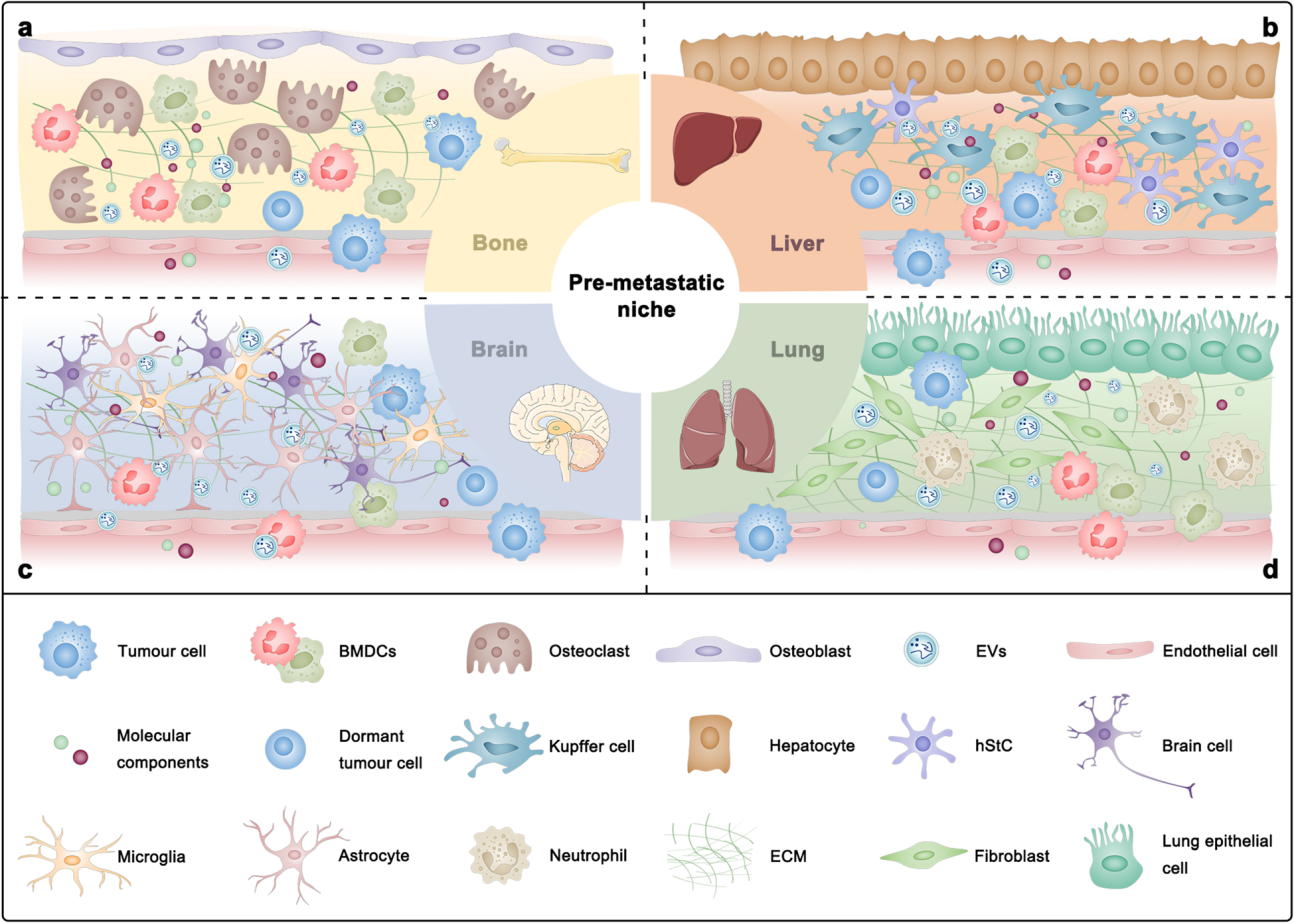
Different characteristics of organ-specific microenvironments determine metastatic organotropism. **a** Bone tropic metastasis. The bone microenvironment formed by osteoblasts, osteoclasts, or other cells promotes bone-specific metastasis. **b** Liver tropic metastasis. Hepatic metastasis is determined by the interactions between tumour cells and different resident subpopulations, including hepatocytes, hepatic stellate cells (hStCs) and Kupffer cells. **c** Brain tropic metastasis. Tumour cells colonizing the brain need to overcome the defence provided by the blood-brain barrier (BBB) and immune cells including astrocytes and microglia. **d** Lung tropic metastasis. Tumour cells can reprogram lung stromal cells, including lung fibroblasts and epithelial cells, which in turn contributes to pulmonary metastasis

Metastasis formation in vital organs is a fatal step of cancer progression and the leading cause of cancer-related mortality. Increasing clinical evidence reveals that certain tumour types preferentially form metastases to survive and outgrow in a number of organs. The commonly targeted organs of metastasis including bone, liver, brain, and lung have been reported to have their own specific PMNs [225] (Fig. 2).

#### Bone

Bone is the third most common site of metastasis for many types of solid tumours, among which breast and prostate cancer are the primary ones [226–228]. Beneficial interactions between tumour cells and various constituents of bone tissue can predispose bone tissues to subsequent tumour colonization and outgrowth [150]. The RANK/RANKL system is a major regulator in the formation of osteolytic bone metastasis [229]. In prostate cancer, RANK-mediated signalling establishes a PMN through a feed-forward loop, involving the induction of RANKL and c-Met, thus promotes cancer cells to colonize the bone [66]. MAOA provides prostate cancer cells with cell growth advantages in the bone microenvironment by stimulating IL-6 release from osteoblasts and triggers skeletal colonization by activating osteoclastogenesis through osteoblast production of RANKL [67]. In breast cancer, T cell-derived RANKL promotes tumour spread and assists bone metastases establishment [230]. Additionally, osteoclastogenesis can be driven by LOX independently of RANKL, leading to osteolytic lesions within the bone microenvironment that support the colonization of tumour cells and the formation of overt bone metastases [75]. Adhesion molecules and CXCR4/CXCL12 chemokine signalling also play an important role in the initiation of bone metastases. In prostate cancer, the CXCL12/CXCR4 axis is crucial for the initial establishment of bone metastases in the endosteal niche, which is severely compromised by the blockade of the CXCL12/CXCR4 axis [231]. Dormant breast cancer cells reside in specific bone marrow niches that regulate their entry into the bone marrow via E-selectin, while anchoring them to the microenvironment via CXCR4/CXCL12 axis [232]. Furthermore, this concept has also been validated in an osteogenic niche, where niche interactions, mediated by cancer-derived E-cadherin and osteogenic N-cadherin, promote early-stage bone colonization of disseminated breast cancer cells [233]. In osteolytic bone metastasis of breast cancer, aberrant expression of VCAM-1, in part dependent on the activity of the NF-κB pathway, promotes the transition from indolent micrometastasis to overt metastasis [234]. Additionally, a balance between the expression of Axl and Tyro3 is associated with a molecular switch between a dormant and a proliferative phenotype of prostate cancer in bone metastases [235].

#### Liver

The liver is the main site of metastatic disease and its infestation is a major cause of death following gastrointestinal malignancies, such as colon, gastric, and pancreatic carcinomas as well as melanoma, breast cancer, and sarcomas [236]. The liver receives a dual blood supply from the hepatic portal vein and hepatic arteries and has a much lower sinusoid blood pressure gradient, which allows CTC access and facilitates their attachment to the sinusoidal endothelium for seeding [225]. Both recruitment of non-resident cells and coordination of liver resident cells are implicated in establishing a supportive liver microenvironment for upcoming tumour cell outgrowth. Epidermal growth factor receptor (EGFR), in exosomes secreted from gastric cancer cells, can be delivered into the pre-metastatic liver and is integrated on the plasma membrane of liver stromal cells including Kupffer cells and hStCs, which effectively triggers hepatotropic metastasis by facilitating the landing and proliferation of metastatic cancer cells [12]. PDAC-derived exosomes that highly express macrophage migration inhibitory factor induce TGF-β secretion by Kupffer cells, which, in turn, promotes fibronectin production by hStCs. Fibronectin deposits subsequently promote the arrest of bone marrow-derived macrophages and neutrophils in the liver, initiating the formation of the PMN [11]. The activation of hStCs, as well as subsequent ECM remodelling, was also reported in the pre-metastatic liver of lung cancer [38]. The homing of integrin α_v_β_5_-expressing exosomes to fibronectin-rich liver microenvironments stimulates Kupffer cells to produce proinflammatory S100A8 and S100P implicated in facilitating tumour metastasis [14]. Furthermore, other proinflammatory cytokines such as IL-6 were found to be released by Kupffer cells influenced by tumour-derived EVs and in association with liver metastasis of colorectal cancer [13]. VEGF-A secreted by colorectal carcinoma cells stimulates TAMs to produce CXCL1 that recruits CXCR2^+^ granulocytic MDSCs to form a PMN that promotes liver metastases. Importantly, liver infiltrating granulocytic MDSCs are able to promote tumour cell survival without the involvement of innate or adaptive immune responses [45]. High systemic TIMP-1 levels increase the liver susceptibility to metastasis by triggering the formation of a PMN through activation of hStCs and recruitment of neutrophils [49, 50]. It has been shown that hepatocytes coordinate myeloid cell accumulation and fibrosis within the liver and, in doing so, increase the susceptibility of the liver to metastatic seeding and outgrowth [69]. Moreover, the adherence of CTCs to fibronectin deposited on the luminal side of liver blood vessels influences the metastatic colonization process from the bloodstream and facilitates liver metastasis of colorectal cancer [237].

#### Brain

Brain metastasis most commonly arises from of lung cancer, breast cancer and melanoma [238]. The blood-brain barrier (BBB) regulates the homeostasis of the central nervous system by forming a tightly regulated neurovascular unit that includes endothelial cells, pericytes and astrocytic end-feet, which together maintain normal brain function [239]. Tumour cell interactions with the brain microenvironment determine organotropic metastasis through various mechanisms including governing cancer-specific signalling pathways involved in metastatic growth. Brain metastatic tumour cells can transfer the second messenger cGAMP to astrocytes via establishing carcinoma-astrocyte gap junctions, activating the STING pathway and production of inflammatory cytokines such as IFN-α and TNF, which activate the STAT1 and NF-κB signalling in brain metastatic cells, thereby supporting tumour growth and chemoresistance [240]. In addition to direct contact, a contact-independent communication mediated by exosomes between metastatic tumour cells and astrocytes was also reported to prime the successful outgrowth of breast cancer cells to form life-threatening metastases [39]. Brain metastatic cells are capable of triggering a phenotypic switch from normal astrocytes to tumour-associated astrocytes through the IL-1β-mediated NF-κB pathway, which, in turn, induces c-Met activation in tumour cells through the expression of hepatocellular growth factor (HGF), promoting survival and growth of brain metastatic cells [241]. Exosomal miR-503 derived from tumour cells promotes M1-M2 conversion of microglia through manipulating STAT3 and NF-κB signalling pathways, followed by enhancing their PD-L1 expression to suppress local immunity and thereby promote brain metastases of breast cancer [25]. The main property of a pre-metastatic brain also lies in BBB destruction that predisposes the brain as a metastatic target. Breast cancer cells can release EVs containing microRNAs such as miR-181c and miR-105 to disrupt BBB integrity by facilitating tight junction dysfunction, promoting the progression of brain metastasis [33, 35]. Furthermore, BBB disruption was also reported to be induced by exosomal CEMIP protein derived from lung and breast cancer [31]. Cathepsin S specifically mediates BBB transmigration of metastatic tumour cells through proteolytic processing of the junctional adhesion molecule, JAM-B [242]. A recent study reports that downregulation of SERPINB1, a protein elevated in brain metastases, led to a reduction in brain metastasis, suggesting that some niche-specific ECM proteins are also involved in metastatic tropism [243]. Additionally, a metastatic tropism analysis showed that intrinsic molecular features of metastatic precursors amongst CTCs can dictate organotropism of metastasis and identified semaphorin 4D as a regulator of tumour cell transmigration through the BBB and MYC as a crucial regulator for the adaptation of DTCs to the activated brain microenvironment [244].

#### Lung

The lung is another organ frequently metastasized by solid tumours such as liver and breast tumours, melanoma, and thyroid tumours [225]. Changes in lung microenvironment in response to a primary tumour can support the survival and outgrowth of metastatic tumour cells. Exosomes carrying integrins α_6_β_4_ and α_6_β_1_ preferentially accumulate in laminin-rich lung microenvironments, where they enhance S100A4 expression in fibroblasts to establish a proinflammatory PMN and promote lung metastasis [14]. Previous studies also reported that tumour cells could establish a pro-metastatic fibronectin-rich environment via phenotypically modulating perivascular cells in favour of the ensuing lung metastasis [245]. Triple-negative breast cancer types can support their metastatic behaviour through modification of ECM proteins such as fibronectin, tenascin-c and periostin and soluble components including the metastasis-associated proteins CCL7, FGFR4, GM-CSF, MMP3, thrombospondin-1 and VEGF in the lung microenvironment [20]. Moreover, it has been found that highly pro-invasive ECM deposited by lung fibroblasts under the influence of mutp53-exosomes is conducive to the metastatic seeding of tumour cells [21]. The pro-metastatic interaction between primary tumour cells and fibroblasts in the lung was also identified in high-metastatic hepatocellular carcinoma [23] and PDAC [22]. Additionally, lung epithelial cells are critical for initiating neutrophil recruitment and lung metastatic niche formation by sensing tumour exosomal RNAs via TLR3 [24]. Neutrophils recruited in large numbers to the inflamed lungs degranulate and release stored proteases that specifically degrade a potent anti-tumorigenic factor, thrombospondin-1, resulting in lung metastasis formation [246]. CCL2 derived from tumour and stromal cells can facilitate lung metastasis by recruiting myeloid cells to lung microenvironments [29, 41, 43]. LOXL2 secreted by breast cancer cells promotes early lung metastasis by controlling the expression of several cytokines and secreted factors and favouring the mobilization and recruitment of CD11b^+^ myeloid cells to lung microenvironments, without modifying ECM stiffness and collagen organization [247]. PMN-derived SDF-1 contributes to lung colonisation by hepatocellular CTCs [53].

Above all, several determinants of organotropic metastasis such as those involved in the interaction between tumour cells and organ-specific resident cells are only relevant to metastasis in correspondence with particular organs. However, organotropic determinants are not necessarily restricted to the one particular organ, due to similar constituents in different PMNs. For instance, the interaction mediated by co-expressed CD146 and CXCR4/CXCL12 signalling between melanoma cancer cells and resident MSCs/pericytes at the perivascular space regulates the extravasation of melanoma cancer cells to bone marrow and liver [52]. Primary tumours can activate resident fibroblasts both in lung and liver, which promote metastatic cancer growth by secreting proinflammatory cytokines such as IL-6 and IL-8 [15].

Additionally, organ-specific metastasis can also be regulated by intrinsic properties of tumour cells. For example, metastatic organotropism of PDAC is dependent upon epithelial plasticity under the governance of p120-catenin. The expression of p120-catenin is required for liver metastasis, while a lack of p120-catenin significantly shifts the metastatic organotropism to the lungs [248]. Furthermore, in human colorectal cancers, CD110^+^ and CDCP1^+^ subpopulations were observed to mediate hepatic and pulmonary metastasis, respectively. CD110^+^ CSCs promote the formation of liver metastases and CDCP1 promotes adhesion of cancer cells to the lung endothelium [249]. In conclusion, PMNs in different target organs displaying distinct molecular and cellular components contribute to the organotropic features of cancer metastasis. Underlying mechanisms need to be elucidated further.

## Targeting the PMN for cancer therapeutics

**Fig. 3:**
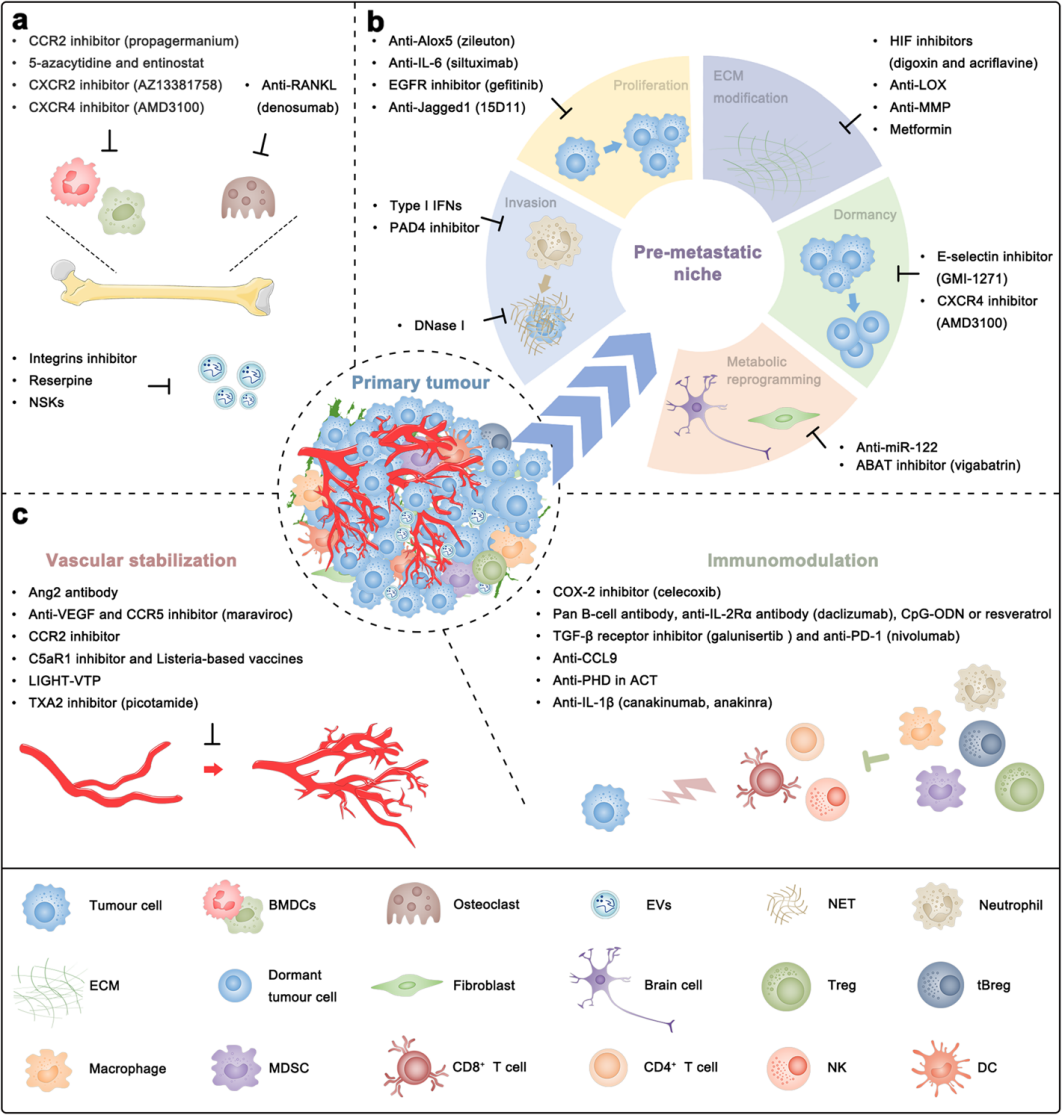
Therapeutic opportunities targeting the pre-metastatic niche (PMN). **a** Targeting extracellular vesicles (EVs) and bone marrow prevents PMN formation. **b** Targeting the interactions between tumour cell and PMN by which tumour cells acquire the ability to successfully develop into metastasis lesions at metastasis sites averts tumour metastasis progression. **c** Targeting vascular destabilization and immunosuppression deconstructs the complexity of the PMN

Tumour metastasis remains the greatest challenge in cancer therapy. Given the importance of the PMN for the regulation of cancer metastasis, targeting various components involved in PMN formation and evolution might represent a promising therapeutic strategy against metastasis (Fig. 3). Therapeutic strategies evaluated and under evaluation in humans are summarized in Table 1.

**Table 1:** Therapeutic strategies against pre-metastatic niche in clinic

Therapeutic Agent	Description	Phase	Cancer Type	Outcomes	Clinical Trials	References
Cilengitide	Integrins (α_v_β_3_ and α_v_β_5_) inhibitor	3	Glioblastoma	The addition of cilengitide to temozolomide chemoradiotherapy did not improve overall survival or progression-free survival	NCT00689221	[250]
Propagermanium	CCR2 inhibitor	1	Breast cancer	Propagermanium proved to be safe as an anti-metastatic drug	UMIN000022494	[251]
5-azacytidine and entinostat	DNA methyltransferase and histone deacetylase inhibitors	1/2	Lung cancer	One complete response and one partial response were observed	NCT00387465	[252]
		2	Breast cancer	One partial response was observed	NCT01349959	[253]
		2	Colorectal cancer	Combination epigenetic therapy proved to be well tolerated but without significant clinical activity	Unknown	[254]
		2	Lung cancer	Under evaluation	NCT01928576	N/A
Plerixafor	CXCR4 inhibitor	2	Pancreatic cancer	Under evaluation	NCT04177810	N/A
Denosumab	Anti-RANKL	3	Breast cancer	Denosumab did not improve bone metastasis-free survival	NCT01077154	[255]
		3	Breast cancer	Denosumab reduced the risk of clinical fractures and improved disease-free survival	NCT00556374	[256]
		3	Prostate cancer	Denosumab delayed initial bone metastases	NCT00286091	[257]
Siltuximab	Anti-IL-6	1/2	Solid tumours	Siltuximab proved to be well tolerated but without clinical activity	Unknown	[258]
Simtuzumab	LOXL2 antibody	2	Colorectal cancer	The addition of simtuzumab to FOLFIRI did not improve progression-free survival, overall survival or objective response rate	NCT01479465	[259]
		2	Pancreatic cancer	The addition of simtuzumab to gemcitabine did not improve progression-free survival, overall survival or objective response rate	NCT01472198	[260]
Andecaliximab	Anti-MMP9	3	Gastric cancer	The addition of andecaliximab to mFOLFOX6 did not improve overall survival	NCT02545504	[261]
Maraviroc	CCR5 blocker	1	Colorectal cancer	Anti-tumoral effects were observed at the tissue level	NCT01736813	[262]
		1	Colorectal cancer	Under evaluation	NCT03274804	N/A
Galunisertib	TGF-β receptor inhibitor	1b/2	Pancreatic cancer	The addition of galunisertib to gemcitabine improved overall survival with minimal added toxicity	NCT01373164	[263]
		1b/2	Solid tumours	Under evaluation	NCT02423343	N/A
Canakinumab	IL-1β inhibitor	3	Lung cancer	Under evaluation	NCT03631199	N/A

*N/A* Not applicable, *RANKL* Receptor activator of NF-κB ligand, *LOXL* Lysyl oxidase-like, *MMP* Matrix metalloproteinases, *TGF-β* Transforming growth factor-β

### Prevention of PMN formation

The components involved in the dynamic process of PMN formation may be potential therapeutic targets to inhibit the formation and function of the PMN, which may be effective in controlling cancer metastasis (Fig. 3a).

The strategy for targeting EVs is widely applicable to tumours in different organs and of different subtypes, essentially because EVs are secreted from nearly all cells, while tumour suppressors have rather narrow applicability because the intrinsic mechanism of cancer metastasis seems to depend on the cancer type. In in vitro and in vivo experiments performed in a mouse model of breast cancer, tumour-derived EVs incubated with the human-specific anti-CD9 or anti-CD63 antibodies were eliminated by macrophages, which significantly decreased metastasis to the lungs, lymph nodes, and thoracic cavity, although no obvious effects on primary xenograft tumour growth were observed [264]. However, the anti-human CD9 and CD63 antibodies are not suitable for direct use in human due to the severe side effects. Targeting exosomal integrins α_6_β_4_ (mediating lung tropism metastasis) and α_v_β_5_ (mediating liver tropism metastasis) may effectively block the uptake of exosomes by resident cells and the subsequent organ-specific metastasis [14]. The effect of cilengitide, a selective inhibitor of α_v_β_3_ and α_v_β_5_ integrins, combined with temozolomide chemoradiotherapy in patients with newly diagnosed glioblastoma with methylated *MGMT* promoter, was assessed in a phase 3 trial (NCT00689221). However, the addition of cilengitide to temozolomide chemoradiotherapy, unfortunately, did not improve outcomes [250]. This failure might be attributed to the reason that the progression of brain tumours, including glioblastomas, generally does not involve distant organs metastasis. Additionally, a preclinical study showed that an anti-hypertensive drug, reserpine, suppressed tumour-derived EVs uptake and disrupted EV-induced formation of the PMN and melanoma lung metastases, which indicates a great potency of reserpine as an adjuvant therapeutic agent in the context of melanoma therapy [265]. Apart from conventional therapeutic drugs, gold nanocages embellished with the platelet and neutrophil hybrid cell membrane, defined as nanosponges and nanokillers (NSKs), can simultaneously capture and clear the CTCs and tumour-derived exosomes via high-affinity membrane adhesion receptors, effectively cutting off the connection between exosomes and immune cells [266]. In addition, the delivery of drugs via exosome membrane packaged core-shell nanoparticles is a promising clinical strategy for cancer prevention and therapy as well. For example, exosome membrane-based nanoparticles can facilitate the delivery of therapeutic siS100A4 to the pre-metastatic lungs via exosome membrane-mediated organotropism and, meanwhile, protect siRNA from enzymatic degradation, mediating efficient cancer prevention [267].

Targeting chemokine signalling represents a potential therapeutic strategy against tumour metastasis. CCL2 seems to be a potent therapeutic target in preventing PMN formation due to its important role in the recruitment of BMDCs via binding CCR2 [41]. However, CCL2 has also been reported to be a critical mediator of optimal anti-metastatic entrainment of G-CSF-stimulated neutrophils, and this type of tumour-entrained neutrophils inhibits tumour cell seeding at a distant pre-metastatic site by generating H_2_O_2_ [174]. Although neutrophils express CCR2, the possibility of neutrophil entrainment through CC receptors other than CCR2 cannot be excluded. The interruption of CCL2 inhibition leads to an overshoot of metastases and accelerates death due to monocyte release from the bone marrow and enhancement of tumour cell mobilization from the primary site, as well as blood vessel formation and increased proliferation of metastatic tumour cells in the lungs in an IL-6- and VEGF-A-dependent manner [268]. These findings indicate that targeting CCL2, which simultaneously possesses both pro-metastatic and anti-metastatic effects due to different receptors on target cells of different types, may be ineffective in preventing PMN formation and tumour metastasis. Therefore, targeting CCR2 might prove a rational approach for preventing cancer metastasis. Propagermanium, a CCR2 antagonist which is currently administered clinically for the treatment of individuals with a hepatitis B virus infection, was shown to have a marked inhibitory effect on cancer metastasis [269]. A phase 1 dose-escalation study indicated an acceptable safety profile of propagermanium used as an anti-metastatic drug for breast cancer patients [251]. Recently, a preclinical study revealed that adjuvant epigenetic therapy using low-dose DNA methyltransferase and histone deacetylase inhibitors, 5-azacytidine and entinostat, disrupted the PMN by inhibiting the trafficking of both monocytic and granulocytic MDSCs through the downregulation of CCR2 and CXCR2, and by promoting monocytic MDSC differentiation into a more-interstitial macrophage-like phenotype [270]. Such epigenetic therapy alone has been reported with limited therapeutic value in certain metastatic cancers, including lung, breast and colorectal cancer [252–254, 271]. Its priming role in anti-PD1 treatment is under evaluation in a phase 2 clinical trial for non-small cell lung cancer (NCT01928576). Another preclinical experiment related to PDAC demonstrated that AZ13381758, a small-molecule inhibitor of CXCR2 that is related to AZD5069, prevented PMN formation by inhibiting BMDC recruitment and improved T cell infiltration into established metastases [272]. Therefore, CXCR2 inhibition in combination with anti-PD-1 immunotherapy may provide hope for effectively targeting multiple stages of the metastatic process in PDAC. Furthermore, pharmacological inhibition of CXCR4 with AMD3100 was reported to reduce TIMP-1-induced PMN formation and neutrophil infiltration without affecting homeostasis of the hematopoietic system [49]. Targeting CXCR4 is not only important for preventing PMN formation via reducing BMDC recruitment but can act in concert with immunotherapies to confine the growth of established metastases. Plerixafor (AMD3100) treatment was shown to reduce fibrosis, alleviate immunosuppression, and significantly enhance the efficacy of immune checkpoint blockers in preclinical models of metastatic breast cancers [273]. The safety and clinical activity of plerixafor in combination with cemiplimab (anti-PD-1) is under evaluation in a phase 2 clinical trial for metastatic pancreatic cancer (NCT04177810). Concluding the above, we propose that targeting specific chemokine receptors on non-tumour cells but not their ligands may hold great promise in the development of effective therapeutic strategies to prevent PMN formation and ensuing tumour metastasis.

The RANK/RANKL signalling, as discussed before, induces an osteolytic and immunosuppressive PMN in bone via the induction of osteoclastogenesis [66] and Treg expansion [46], which suggests targeting RANK/RANKL will potentially be effective in preventing PMN formation and bone metastasis by tumours. Denosumab is a human monoclonal antibody that targets the RANKL, with high affinity and specificity for the soluble and cell membrane-bound forms of human RANKL [274]. In the latest double-blind, randomised, placebo-controlled, phase 3 trial (NCT01077154), the effect of denosumab in combination with adjuvant or neoadjuvant systemic therapy in women with histologically confirmed stage II or III breast cancer was assessed [255]. Results for the primary endpoint of bone metastasis-free survival uncovered that denosumab did not improve disease-related outcomes for women with high-risk early breast cancer. However, a previous phase 3 trial (NCT00556374) showed that treatment with adjuvant denosumab reduced the risk of clinical fractures and improved disease-related outcomes in patients with postmenopausal hormone receptor-positive early breast cancer receiving aromatase inhibitor therapy [256]. Moreover, denosumab was also tested in another phase 3 trial (NCT00286091) and was shown to delay initial bone metastases in patients with castration-resistant prostate cancer [257]. In summary, although the exact effect of denosumab in bone metastasis disease should be identified in future studies, denosumab does indicate that the RANK/RANKL signalling pathway, as an important signalling pathway for the establishment of the PMN in bone, can be targeted to improve the bone metastasis-related outcomes of patients with aggressive tumours.

### Targeting the interaction between tumour cell and PMN

The successful extravasation, colonization and outgrowth of metastatic tumour cells in PMN is a severe rate-limiting step during metastasis progression [275]. Targeting the interactions between tumour cells and PMN may be a novel therapeutic strategy to avert tumour metastasis progression (Fig. 3b).

NETs formed by neutrophils in the PMN can promote metastatic colonization of tumour cells [276, 277]. Therefore, inhibiting NET formation or digesting NETs with deoxyribonuclease I (DNase I) may block metastatic progression. DNase I is approved by the FDA for the treatment of cystic fibrosis, in which it decreases mucus viscosity, resulting from NET accumulation and triggered by persistent infections. DNase I-coated nanoparticles overcome the weakness of short half-life in blood and inhibit tumour metastasis, which serves as proof that NETs are possible drug targets to reduce metastasis [278]. Another strategy to avert NET formation is using a PAD4 pharmacologic inhibitor that has been testified efficient in preventing omental metastasis in a preclinical experiment [276]. Furthermore, NETs can also shield tumour cells from cytotoxicity, as mediated by CD8^+^ T cells and NK cells, by obstructing close contact with these cytotoxic immune cells [279], indicating that the combination of NET blockade with immunotherapies, such as immune checkpoint inhibitors, represents a promising strategy to prevent metastatic progression. In addition to NETs, leukotrienes derived from tumour-mobilized lung neutrophils aid the tumour colonization of distant tissues by selectively increasing the proliferation of metastasis-initiating tumour cells in a pERK1/2-dependent manner, and Zileuton, an inhibitor of leukotriene-generating enzyme Alox5, abrogates neutrophil pro-metastatic activity and consequently reduces metastasis [175]. It was preclinically demonstrated that endogenous type I IFNs regulated the cellular composition within the PMN by restricting migration of neutrophils and therefore reduced the expression of pro-metastatic molecules such as S100A8, S100A9, Bv8 and MMP9 and inhibited extravasation efficiency of tumour cells [280]. Therefore, the application of low dose type I IFNs in the perioperative period could be a promising approach reducing the risk of metastases development, especially in patients with impaired type I IFN signalling.

IL-6 derived from PMN endows metastatic tumour cells with proliferative advantages [67, 68, 281] and the resistance to cytotoxic T cells through the overexpression of immunosuppressive molecules like PD-L1 [282], which suggests that IL-6 is a promising target for both restriction of tumour proliferation and restoration of host immunity. Siltuximab, a monoclonal antibody with high binding affinity for human IL-6, was reported in a phase 1/2 trial to be well tolerated but without clinical activity in solid tumours including ovarian and *KRAS* mutant cancers [258]. Interestingly, a recent study showed that treatment of ovarian cancer cells with neutralizing IL-6 antibodies resulted in upregulated EGFR, whereas the combination of neutralizing IL-6 antibodies and the EGFR inhibitor gefitinib exhibited enhanced anticancer activity [283]. Moreover, EGFR in exosomes secreted from gastric cancer cells can be delivered into the pre-metastatic liver and is integrated on the plasma membrane of liver stromal cells, including Kupffer cells and hStCs, which effectively facilitates the landing and proliferation of metastatic cancer cells [12]. Collectively, it is expected that a combination of IL-6-targeted therapy with EGFR inhibitor gefitinib or other therapies, such as chemo- or immunotherapy like anti-PD-L1, will be more efficient than monotherapy. Although chemotherapy is cytotoxic to tumour cells, its anti-metastatic potential might be compromised by the stromal cells in the PMN. For example, osteoblast-derived Jagged1 activates Notch signalling in tumour cells to promote resistance to chemotherapy-induced apoptosis. The combination of chemotherapy with 15D11, a fully human monoclonal antibody against Jagged1, may nullify such stroma-mediated mechanism of chemoresistance and help achieve optimal outcomes in the treatment of bone metastasis [284].

LOX family proteins, induced mainly by hypoxia, promote PMN evolution and ensuing tumour colonization and proliferation [73–77]. As a result, these hypoxia-induced enzymes are potent mediators of tumour metastasis and promising novel therapeutic targets. Two chemically and mechanistically distinct HIF inhibitors, digoxin and acriflavine, were reported to hinder PMN evolution by blocking the hypoxia-induced expression of LOX family proteins, collagen cross-linking and CD11b^+^ BMDC recruitment, thus preventing lung metastasis in an orthotopic breast cancer model [285]. Therefore, both digoxin and acriflavine are suitable candidates for clinical trials for breast cancer, especially in those patients whose primary tumours express high levels of HIF-1α. Moreover, inhibition of LOX was demonstrated to lead to a decrease in collagen crosslinking and subsequently a reduction in the generation of the insoluble fibrotic matrix, thus preventing breast cancer metastasis [286]. However, another animal experiment proved that treatment with β-aminopropionitrile (BAPN), a LOX inhibitor, did not suppress the lymph node metastases [287]. LOXL2 was shown to promote fibronectin production, MMP9 and CXCL12 expression and BMDCs recruitment to assist PMN formation [288], whereas a randomized phase 2 trials showed that simtuzumab, an antibody to LOXL2, did not improve clinical outcomes in patients with metastatic *KRAS* mutant colorectal carcinoma [259] or metastatic pancreatic adenocarcinoma [260]. These seemingly paradoxical outcomes in preclinical studies and clinical trials may be related to the extremely selective nature of specific antibodies that lack cross-inhibition of other LOX family proteins. MMPs were shown to be responsible for ECM modification and vascular destabilization in PMN [51, 74, 152, 289]. The efficacy and safety of andecaliximab, a monoclonal antibody that inhibits MMP9, combined with mFOLFOX6 as first-line treatment in patients with advanced gastric or gastroesophageal junction adenocarcinoma was evaluated in a phase 3 clinical trial [261]. Regrettably, the results indicated that the addition of andecaliximab to mFOLFOX6 did not improve the overall survival of these patients. Further investigations are needed to explore the exact role of targeting LOX family proteins and MMPs during tumour metastasis and to understand the circumstances under which targeting ECM remodelling may be used as a therapeutic strategy for cancer patients. Additionally, metformin was recently reported to prevent the development of a fibrotic PMN [290], indicating its potential use for precaution against tumour metastasis.

Another major problem in the clinical management of tumour is metastatic dormancy, as it presents a risk of relapse and undermines the benefit of current adjuvant chemotherapies and hormonal therapies as well. Therefore, targeting metastatic tumour dormancy represents a possible way of therapeutically intervening metastasis. A preclinical study demonstrated that GMI-1271, a highly specific glycomimetic E-selectin binding inhibitor, blocked E-selectin on the lumen of specialized vascular beds in the bone marrow, preventing vessel adhesion and subsequent passage of circulating breast cancer cells into the tissue, and that AMD3100 (a CXCR4 inhibitor) forced dormant breast cancer cells, inhabiting these same perivascular niches, into the bloodstream [232]. Simultaneous blockade of CXCR4 and E-selectin could exclude dormant metastatic cancer cells from the protective bone marrow environment and block their re-entry into niches, preventing recurrence. Additionally, E-selectin antagonists were also shown to prevent hematogenous metastasis of breast cancer via the inhibition of a shear-resistant adhesion of cancer cells to E-selectin-expressing blood vessels on the PMN [291, 292].

Targeting the metabolic changes in the PMN may also be a promising therapeutic strategy to prevent tumour metastasis. Breast cancer-secreted miR-122 was reported to suppress glucose uptake by non-tumour cells in the PMN through downregulating the glycolytic enzyme pyruvate kinase, which allowed metastasizing breast cancer cells to accommodate their nutrient requirements of glucose in the PMN and facilitates metastatic seeding [19]. Accordingly, miR-122-targeted therapy in metastatic cancer patients seems highly feasible, and the non-invasive blood test for circulating miR-122 would enable accurate selection of patients who may benefit from this treatment. Moreover, the occurrence of neural niches may allow the use of various available neurotransmitter modulators as metabolically related anti-metastatic drugs. For instance, vigabatrin, an irreversible inhibitor of γ-Aminobutyric acid (GABA) transaminase (ABAT), was experimentally shown to prevent brain metastasis by breast cancer cells by avoiding GABA catabolism [293]. In addition, an artificial PMN fabricated by embedding exosomes onto a 3D scaffold was indicated to be an effective approach to disrupt the process of metastasis through capturing metastatic cells disseminating in the peritoneal cavity [294]. This research suggests that a potent artificial PMN is an effective approach to impair the metastatic cells colonization of the PMN, representing a disruptive technology that complements current surgical and chemotherapeutic approaches in advanced gynaecological and gastrointestinal malignancies with a peritoneal dissemination pattern.

### Deconstructing the complexity of the PMN

Vascular destabilization and immunosuppression, which are major principal characteristics of the PMN, enable the PMN to be suitable for tumour cell extravasation, colonization and proliferation. Targeting these characteristics to deconstruct the complexity of the PMN may be a very promising strategy for metastasis therapeutics (Fig. 3c).

#### Vascular stabilization

Vascular destabilization is crucial for the extravasation of tumour cells and subsequent metastasis and is reflected in many aspects including high vascular permeability, angiogenesis and lymphangiogenesis in the PMN. Ang2 that promotes angiogenesis is highly upregulated in the PMN [62, 152], indicating that it may act as a potential therapeutic target in tumour metastasis. Preclinical data demonstrated that inhibition of Ang2 following primary tumour resection significantly reduced the metastatic burden and exerted an antiangiogenic response with stabilized residual vasculature in metastases, and that combining Ang2 antibody with low-dose metronomic chemotherapy interfering with the recruitment of the anti-VEGF resistance-conferring myeloid cells further improved the therapeutic benefit with fewer adverse effects than high-dose chemotherapy [295]. Moreover, anti-angiogenic therapies, such as anti-VEGF therapy, were shown to be in synergy with the FDA-approved anti-retroviral drug, maraviroc (CCR5 blocker), which is orally available and safe for long-term use, giving rise to further possibilities of the therapeutic intervention for metastatic breast cancer [64]. The anti-tumoral effect of maraviroc was confirmed at the tissue level in patients with liver metastases of advanced refractory colorectal cancer [262] and is, currently, evaluated in a phase 1 clinical trial for metastatic colorectal cancer (NCT03274804). Additionally, VCAM-1 binding peptide tagged liposomes carrying the CCR2 antagonist can reduce pre-metastatic lung vascular permeability by directly targeting cancer cell-activated endothelium, and thereby prevent tumour cell extravasation [296]. A separate preclinical study proved that inhibition of C5aR1 in combination with antiangiogenic *Listeria*-based vaccines reduced or prevented breast cancer metastasis by reducing vascular density and improving antitumor immunity in the pre-metastatic lungs, and that this therapeutic approach was more efficacious in reducing lung metastatic burden than sunitinib, a small-molecule receptor tyrosine kinase inhibitor that has well-established antiangiogenic properties resulting from its interactions with PDGFR and VEGFR [297]. This combinational strategy has the potential to overcome the resistance to antiangiogenic monotherapies targeting exclusively the VEGF pathway because poor responses to these therapies are driven by the upregulation of alternative proangiogenic pathways, increased protective pericyte coverage of tumour blood vessels and the recruitment of proangiogenic myeloid inflammatory cells, including MDSCs to tumours [297].

Apart from reducing vascular density, vessel normalization seems to be another effective strategy to reverse vascular destabilization in the PMN. LIGHT-VTP, which consists of the TNF superfamily member LIGHT or TNFSF14 and a vascular targeting peptide (VTP) that specifically binds to angiogenic tumour vasculature, efficiently targets pathological blood vessels in the PMN, reducing vascular hyper-permeability and ECM deposition, thus blocking metastatic lung colonization [298]. Moreover, LIGHT-VTP decreases intravasation of tumour cells from the primary sites into the circulation, and also reverses vessel abnormalities and sensitizes tumours for checkpoint inhibitor antibodies against the PD-1 once metastatic nodules have formed [298]. These combinational strategies offer a new therapeutic avenue for the treatment of aggressive metastatic cancers unresponsive to the current therapies. Additionally, preclinical data identified that aspirin, an inhibitor of both cyclooxygenase (COX) isoforms, prevented the formation of an intravascular metastasis and PMN through inhibition of platelet-derived thromboxane A_2_ (TXA_2_) [299]. However, aspirin significantly increases the risk of severe gastrointestinal symptoms and complications, especially over long-term use. Therefore, selective TXA_2_ inhibitors such as picotamide might present an alternative to target platelet-derived TXA_2_ while sparing other gastroprotective COX-1 products (i.e., prostacyclin), and thus might be a safer therapeutic option for the prevention of metastatic disease [299].

#### Immunomodulation

Deconstructing immunosuppression is a promising therapeutic strategy to inhibit the pro-metastatic properties of the PMN. COX-2-derived PGE_2_ has been identified as a crucial factor for the accumulation of immunosuppressive cells in the PMN [48, 300]. As a result, it should be targeted to improve the anti-tumour response. Preclinical studies demonstrated that celecoxib, a COX-2 inhibitor, could inhibit the infiltration of immunosuppressive cells in the PMNs and subsequent tumour metastasis in lymph nodes [48] and brain [300]. In another experiment, celecoxib was shown to inhibit lymph node metastasis of breast cancer by cutting off the link between B cell-derived pathogenic IgG and SDF1α secretion by lymph node stromal cells [301]. Tumour-evoked regulatory B cells (tBregs) that are actively generated by normal B cells in response to the direct effects of tumour-derived factors, induce the conversion of metastasis-supporting FoxP3^+^ Tregs from nonregulatory CD4^+^ T cells [302] and activate the regulatory function of both the monocyte and granulocyte subpopulations of MDSCs [303], both of which depend on TGF-β signalling, thus inducing an immunosuppressive environment in PMN to support lung metastasis. As a result, tBregs need to be targeted to reduce the expansion and activation of immunosuppressive cell populations such as Tregs and MDSCs, enhancing antitumor immune responses. Clinically available antibodies such as the pan B-cell antibody, anti-CD20 antibody (rituximab) or the anti-IL-2Rα antibody (daclizumab) could bypass the tBreg-mediated blockade of the immune response to some cancers [302]. However, it was preclinically shown that rituximab did not provide benefits in solid tumours, as it enriches for CD20^Low^ tBregs via depletion of the beneficial antitumor CD20^+^ B cells, and thereby further enhances lung metastasis by exacerbating tBreg-mediated immunosuppression [304]. Therefore, tBreg-targeted therapies should avoid depleting beneficial antitumor B cells. CXCL13-coupled CpG oligonucleotides (CpG-ODN) [304] and resveratrol [305] can efficiently inhibit tBregs without nonspecific inactivation of effector immune cells, representing a promising strategy to enhance cancer therapy by targeting tBregs. Besides, the aforementioned studies also provide an important rationale for targeting TGF-β in order to improve anti-tumour immune responses. Galunisertib is the first orally bioavailable small-molecule inhibitor of the type I TGF-β receptor (ALK5) serine/threonine kinase to enter clinical development [306]. In a phase 1b/2 trial (NCT01373164), galunisertib in combination with gemcitabine improved overall survival in patients with unresectable pancreatic cancer, with minimal added toxicity, compared with gemcitabine alone [263]. Targeting both TGF-β and immune checkpoint in combination will be an effective approach to suppress tumour metastasis. Clinical trials are currently exploring the combination of anti-PD-1 (nivolumab) and galunisertib in advanced refractory solid tumours (NCT02423343). CCL9, as a downstream mediator of the metastasis-promoting function of TGF-β signalling in MDSCs, may be a good therapeutic target and might bypass some of the negative consequences of TGF-β neutralization [307].

T-cell-intrinsic expression of the oxygen-sensing prolyl-hydroxylase (PHD) proteins was found to suppress anti-tumour immunity in the pre-metastatic lungs through restraining inflammatory CD4^+^ and CD8^+^ T cell responses and permitting immunosuppressive Treg differentiation [308]. Therefore, inhibition of PHD proteins could offer a viable clinical strategy to limit lung metastasis. Addition of a PHD inhibitor to established clinical expansion protocols for human adoptive cell therapy (ACT) using tumour-infiltrating lymphocytes (TILs) or chimeric antigen receptor (CAR)-transduced T cells is a feasible and potentially effective therapeutic strategy to improve the functional quality of tumour-specific T cells [308]. Tumour-induced neutrophils also acquire the ability to suppress cytotoxic T lymphocytes carrying the CD8 antigen, which limits the establishment of metastases. For example, IL-1β elicits IL-17 expression from γδ T cells, resulting in a systemic G-CSF-dependent expansion and polarization of neutrophils towards a CD8^+^ T cell-suppressive phenotype and subsequent metastasis formation in distant organs [309]. Targeting this novel IL-1β/γδ T cell/IL-17/neutrophil axis represents a new strategy to modulate the immunosuppressive state of the PMN and metastatic disease progression. Additionally, IL-1β was found to promote the recruitment of MDSCs and macrophages via chemokine expression [310] and tumour cell arrest on endothelial cells via E-selectin expression [311] in the PMN. Preclinical data demonstrated that anakinra, IL-1 receptor antagonist, limited metastasis, and MDSC recruitment at early stages of tumour progression but failed to reverse established metastatic tumours [310]. Moreover, blocking IL-1β can synergize with anti-PD-1 for tumour abrogation [312]. The efficacy and safety of pembrolizumab (anti-PD-1) plus platinum-based doublet chemotherapy with or without canakinumab (IL-1β inhibitor) as first-line therapy, is currently evaluated in a randomized, double-blind, placebo-controlled, phase 3 study for subjects with locally advanced or metastatic non-squamous and squamous non-small cell lung cancer (NCT03631199).

## Conclusion

PMN formation is initiated by the dynamic and complex interaction of tumour cells with the surrounding microenvironment in the primary metastatic site. Investigating this interaction has led to a deeper understanding of the mechanisms underlying metastasis, which could potentially aid in developing new therapeutic strategies to hinder metastatic development. Although several therapeutic approaches have already been introduced, it is still necessary to develop other strategies that will target the PMN more effectively. Additionally, highly sensitive and specific biomarkers are required to identify suitable patients that will benefit from these anti-metastatic therapies. The use of animal models and other experimental techniques is needed to further illuminate the biological network within the PMN and will potentially help the clinical translation of niche-targeted approaches. Applying niche-targeted therapies together with other anti-tumour therapies holds great promise for the effective prevention of metastasis.

## Data Availability

Not applicable.

## Abbreviations

BBB: Blood-brain barrier
BMDCs: Bone marrow-derived cells
CAF: Carcinoma-associated fibroblast
COX: Cyclooxygenase
CSCs: Cancer stem cells
CTCs: Circulating tumour cells
DCs: Dendritic cells
DTCs: Disseminated tumour cells
ECM: Extracellular matrix
EGFR: Epidermal growth factor receptor
EVs: Extracellular vesicles
FGF: Fibroblast growth factor
G-CSF: Granulocyte-colony stimulating factor
GAG: Glycosaminoglycan
HA: Hyaluronan
HIF: Hypoxia-inducible factor
HSCs: Hematopoietic stem cells
hStCs: Hepatic stellate cells
IL: Interleukin
LECs: Lymphatic endothelial cells
LOX: Lysyl oxidase
LOXL: Lysyl oxidase-like
MAMs: Metastasis-associated macrophages
MDSCs: Myeloid-derived suppressor cells
MMP: Matrix metalloproteinases
MSCs: Mesenchymal stem cells
NET: Neutrophil extracellular trap
NOX2: NADPH oxidase 2
PD-1: Programmed death-1
PD-L1: Programmed death-ligand 1
PDAC: Pancreatic ductal adenocarcinoma
PMN: Pre-metastatic niche
RANK: Receptor activator of NF-κB
RANKL: Receptor activator of NF-κB ligand
SAA: Serum amyloid A
SDF-1: Stromal-derived factor 1
TAMs: Tumour-associated macrophages
tBregs: Tumour-evoked regulatory B cells
TGF-β: Transforming growth factor-β
TLR: Toll-like receptor
TNF: Tumour necrosis factor
Tregs: Regulatory T cells
VCAM-1: Vascular cell adhesion molecule-1
VEGF: Vascular endothelial growth factor
VEGFR: Vascular endothelial growth factor receptor

## References

[1] Fares J, Fares MY, Khachfe HH, Salhab HA, Fares Y (2020). Molecular principles of metastasis: a hallmark of cancer revisited. Signal Transduct Target Ther.

[2] Liu Y, Cao X (2016). Characteristics and significance of the pre-metastatic niche. Cancer Cell.

[3] Paget S (1889). The distribution of secondary growths in cancer of the breast. Lancet.

[4] Valadi H, Ekström K, Bossios A, Sjöstrand M, Lee JJ, Lötvall JO (2007). Exosome-mediated transfer of mRNAs and microRNAs is a novel mechanism of genetic exchange between cells. Nat Cell Biol.

[5] Skog J, Würdinger T, van Rijn S, Meijer DH, Gainche L, Sena-Esteves M (2008). Glioblastoma microvesicles transport RNA and proteins that promote tumour growth and provide diagnostic biomarkers. Nat Cell Biol.

[6] Théry C, Zitvogel L, Amigorena S (2002). Exosomes: composition, biogenesis and function. Nat Rev Immunol.

[7] Redzic JS, Balaj L, van der Vos KE, Breakefield XO (2014). Extracellular RNA mediates and marks cancer progression. Semin Cancer Biol.

[8] Zhu L, Sun H-T, Wang S, Huang S-L, Zheng Y, Wang C-Q (2020). Isolation and characterization of exosomes for cancer research. J Hematol Oncol.

[9] Guo Y, Ji X, Liu J, Fan D, Zhou Q, Chen C (2019). Effects of exosomes on pre-metastatic niche formation in tumors. Mol Cancer.

[10] Lobb RJ, Lima LG, Möller A (2017). Exosomes: key mediators of metastasis and pre-metastatic niche formation. Semin Cell Dev Biol.

[11] Costa-Silva B, Aiello NM, Ocean AJ, Singh S, Zhang H, Thakur BK (2015). Pancreatic cancer exosomes initiate pre-metastatic niche formation in the liver. Nat Cell Biol.

[12] Zhang H, Deng T, Liu R, Bai M, Zhou L, Wang X (2017). Exosome-delivered EGFR regulates liver microenvironment to promote gastric cancer liver metastasis. Nat Commun.

[13] Shao Y, Chen T, Zheng X, Yang S, Xu K, Chen X (2018). Colorectal cancer-derived small extracellular vesicles establish an inflammatory premetastatic niche in liver metastasis. Carcinogenesis.

[14] Hoshino A, Costa-Silva B, Shen TL, Rodrigues G, Hashimoto A, Tesic Mark M (2015). Tumour exosome integrins determine organotropic metastasis. Nature.

[15] Ji Q, Zhou L, Sui H, Yang L, Wu X, Song Q (2020). Primary tumors release ITGBL1-rich extracellular vesicles to promote distal metastatic tumor growth through fibroblast-niche formation. Nat Commun.

[16] Dai J, Escara-Wilke J, Keller JM, Jung Y, Taichman RS, Pienta KJ (2019). Primary prostate cancer educates bone stroma through exosomal pyruvate kinase M2 to promote bone metastasis. J Exp Med.

[17] Chow A, Zhou W, Liu L, Fong MY, Champer J, Van Haute D (2014). Macrophage immunomodulation by breast cancer-derived exosomes requires toll-like receptor 2-mediated activation of NF-κB. Sci Rep.

[18] Zhang H, Yu Y, Zhou L, Ma J, Tang K, Xu P (2018). Circulating tumor microparticles promote lung metastasis by reprogramming inflammatory and mechanical niches via a macrophage-dependent pathway. Cancer Immunol Res.

[19] Fong MY, Zhou W, Liu L, Alontaga AY, Chandra M, Ashby J (2015). Breast-cancer-secreted miR-122 reprograms glucose metabolism in premetastatic niche to promote metastasis. Nat Cell Biol.

[20] Medeiros B, Goodale D, Postenka C, Lowes LE, Kiser P, Hearn S, et al. Triple-negative primary breast tumors induce supportive premetastatic changes in the extracellular matrix and soluble components of the lung microenvironment. Cancers (Basel). 2020;12(1). 10.3390/cancers12010172. 10.3390/cancers12010172 PMC7016570 31936750

[21] Novo D, Heath N, Mitchell L, Caligiuri G, MacFarlane A, Reijmer D (2018). Mutant p53s generate pro-invasive niches by influencing exosome podocalyxin levels. Nat Commun.

[22] Armacki M, Polaschek S, Waldenmaier M, Morawe M, Ruhland C, Schmid R, et al. Protein kinase D1, reduced in human pancreatic tumors, increases secretion of small extracellular vesicles from cancer cells that promote metastasis to lung in mice. Gastroenterology. 2020. 10.1053/j.gastro.2020.05.052. 10.1053/j.gastro.2020.05.052 32446697

[23] Fang T, Lv H, Lv G, Li T, Wang C, Han Q (2018). Tumor-derived exosomal miR-1247-3p induces cancer-associated fibroblast activation to foster lung metastasis of liver cancer. Nat Commun.

[24] Liu Y, Gu Y, Han Y, Zhang Q, Jiang Z, Zhang X (2016). Tumor exosomal RNAs promote lung pre-metastatic niche formation by activating alveolar epithelial TLR3 to recruit neutrophils. Cancer Cell.

[25] Xing F, Liu Y, Wu SY, Wu K, Sharma S, Mo YY (2018). Loss of XIST in breast cancer activates MSN-c-Met and reprograms microglia via exosomal miRNA to promote brain metastasis. Cancer Res.

[26] Peinado H, Alečković M, Lavotshkin S, Matei I, Costa-Silva B, Moreno-Bueno G (2012). Melanoma exosomes educate bone marrow progenitor cells toward a pro-metastatic phenotype through MET. Nat Med.

[27] Zhao J, Schlößer HA, Wang Z, Qin J, Li J, Popp F, et al. Tumor-derived extracellular vesicles inhibit natural killer cell function in pancreatic cancer. Cancers (Basel). 2019;11(6). 10.3390/cancers11060874. 10.3390/cancers11060874 PMC6628179 31234517

[28] Wen SW, Sceneay J, Lima LG, Wong CS, Becker M, Krumeich S (2016). The biodistribution and immune suppressive effects of breast cancer-derived exosomes. Cancer Res.

[29] Keklikoglou I, Cianciaruso C, Güç E, Squadrito ML, Spring LM, Tazzyman S (2019). Chemotherapy elicits pro-metastatic extracellular vesicles in breast cancer models. Nat Cell Biol.

[30] Yang WW, Yang LQ, Zhao F, Chen CW, Xu LH, Fu J (2017). Epiregulin promotes lung metastasis of salivary adenoid cystic carcinoma. Theranostics.

[31] Rodrigues G, Hoshino A, Kenific CM, Matei IR, Steiner L, Freitas D (2019). Tumour exosomal CEMIP protein promotes cancer cell colonization in brain metastasis. Nat Cell Biol.

[32] Grange C, Tapparo M, Collino F, Vitillo L, Damasco C, Deregibus MC (2011). Microvesicles released from human renal cancer stem cells stimulate angiogenesis and formation of lung premetastatic niche. Cancer Res.

[33] Zhou W, Fong MY, Min Y, Somlo G, Liu L, Palomares MR (2014). Cancer-secreted miR-105 destroys vascular endothelial barriers to promote metastasis. Cancer Cell.

[34] Zeng Z, Li Y, Pan Y, Lan X, Song F, Sun J (2018). Cancer-derived exosomal miR-25-3p promotes pre-metastatic niche formation by inducing vascular permeability and angiogenesis. Nat Commun.

[35] Tominaga N, Kosaka N, Ono M, Katsuda T, Yoshioka Y, Tamura K (2015). Brain metastatic cancer cells release microRNA-181c-containing extracellular vesicles capable of destructing blood-brain barrier. Nat Commun.

[36] Plebanek MP, Angeloni NL, Vinokour E, Li J, Henkin A, Martinez-Marin D (2017). Pre-metastatic cancer exosomes induce immune surveillance by patrolling monocytes at the metastatic niche. Nat Commun.

[37] Kong J, Tian H, Zhang F, Zhang Z, Li J, Liu X (2019). Extracellular vesicles of carcinoma-associated fibroblasts creates a pre-metastatic niche in the lung through activating fibroblasts. Mol Cancer.

[38] Hsu YL, Huang MS, Hung JY, Chang WA, Tsai YM, Pan YC (2020). Bone-marrow-derived cell-released extracellular vesicle miR-92a regulates hepatic pre-metastatic niche in lung cancer. Oncogene.

[39] Zhang L, Zhang S, Yao J, Lowery FJ, Zhang Q, Huang WC (2015). Microenvironment-induced PTEN loss by exosomal microRNA primes brain metastasis outgrowth. Nature.

[40] Ono M, Kosaka N, Tominaga N, Yoshioka Y, Takeshita F, Takahashi RU (2014). Exosomes from bone marrow mesenchymal stem cells contain a microRNA that promotes dormancy in metastatic breast cancer cells. Sci Signal.

[41] Qian BZ, Li J, Zhang H, Kitamura T, Zhang J, Campion LR (2011). CCL2 recruits inflammatory monocytes to facilitate breast-tumour metastasis. Nature.

[42] Wolf MJ, Hoos A, Bauer J, Boettcher S, Knust M, Weber A (2012). Endothelial CCR2 signaling induced by colon carcinoma cells enables extravasation via the JAK2-Stat5 and p38MAPK pathway. Cancer Cell.

[43] Sceneay J, Chow MT, Chen A, Halse HM, Wong CS, Andrews DM (2012). Primary tumor hypoxia recruits CD11b+/Ly6Cmed/Ly6G+ immune suppressor cells and compromises NK cell cytotoxicity in the premetastatic niche. Cancer Res.

[44] van Deventer HW, Palmieri DA, Wu QP, McCook EC, Serody JS (2013). Circulating fibrocytes prepare the lung for cancer metastasis by recruiting Ly-6C+ monocytes via CCL2. J Immunol.

[45] Wang D, Sun H, Wei J, Cen B, DuBois RN (2017). CXCL1 is critical for premetastatic niche formation and metastasis in colorectal cancer. Cancer Res.

[46] Zhao E, Wang L, Dai J, Kryczek I, Wei S, Vatan L (2012). Regulatory T cells in the bone marrow microenvironment in patients with prostate cancer. Oncoimmunology.

[47] Olkhanud PB, Baatar D, Bodogai M, Hakim F, Gress R, Anderson RL (2009). Breast cancer lung metastasis requires expression of chemokine receptor CCR4 and regulatory T cells. Cancer Res.

[48] Ogawa F, Amano H, Eshima K, Ito Y, Matsui Y, Hosono K (2014). Prostanoid induces premetastatic niche in regional lymph nodes. J Clin Invest.

[49] Seubert B, Grünwald B, Kobuch J, Cui H, Schelter F, Schaten S (2015). Tissue inhibitor of metalloproteinases (TIMP)-1 creates a premetastatic niche in the liver through SDF-1/CXCR4-dependent neutrophil recruitment in mice. Hepatology.

[50] Grünwald B, Harant V, Schaten S, Frühschütz M, Spallek R, Höchst B (2016). Pancreatic premalignant lesions secrete tissue inhibitor of metalloproteinases-1, which activates hepatic stellate cells via CD63 signaling to create a premetastatic niche in the liver. Gastroenterology.

[51] Kaplan RN, Riba RD, Zacharoulis S, Bramley AH, Vincent L, Costa C (2005). VEGFR1-positive haematopoietic bone marrow progenitors initiate the pre-metastatic niche. Nature.

[52] Correa D, Somoza RA, Lin P, Schiemann WP, Caplan AI (2016). Mesenchymal stem cells regulate melanoma cancer cells extravasation to bone and liver at their perivascular niche. Int J Cancer.

[53] Tang Y, Lu Y, Chen Y, Luo L, Cai L, Peng B (2019). Pre-metastatic niche triggers SDF-1/CXCR4 axis and promotes organ colonisation by hepatocellular circulating tumour cells via downregulation of Prrx1. J Exp Clin Cancer Res.

[54] Deng J, Liu Y, Lee H, Herrmann A, Zhang W, Zhang C (2012). S1PR1-STAT3 signaling is crucial for myeloid cell colonization at future metastatic sites. Cancer Cell.

[55] Zhang W, Zhang C, Li W, Deng J, Herrmann A, Priceman SJ (2015). CD8+ T-cell immunosurveillance constrains lymphoid premetastatic myeloid cell accumulation. Eur J Immunol.

[56] Vadrevu SK, Chintala NK, Sharma SK, Sharma P, Cleveland C, Riediger L (2014). Complement c5a receptor facilitates cancer metastasis by altering T-cell responses in the metastatic niche. Cancer Res.

[57] Sharma SK, Chintala NK, Vadrevu SK, Patel J, Karbowniczek M, Markiewski MM (2015). Pulmonary alveolar macrophages contribute to the premetastatic niche by suppressing antitumor T cell responses in the lungs. J Immunol.

[58] Wang Z, Xiong S, Mao Y, Chen M, Ma X, Zhou X (2016). Periostin promotes immunosuppressive premetastatic niche formation to facilitate breast tumour metastasis. J Pathol.

[59] Hiratsuka S, Watanabe A, Aburatani H, Maru Y (2006). Tumour-mediated upregulation of chemoattractants and recruitment of myeloid cells predetermines lung metastasis. Nat Cell Biol.

[60] Kim KJ, Kwon SH, Yun JH, Jeong HS, Kim HR, Lee EH (2017). STAT3 activation in endothelial cells is important for tumor metastasis via increased cell adhesion molecule expression. Oncogene.

[61] Wieland E, Rodriguez-Vita J, Liebler SS, Mogler C, Moll I, Herberich SE (2017). Endothelial Notch1 activity facilitates metastasis. Cancer Cell.

[62] Minami T, Jiang S, Schadler K, Suehiro J, Osawa T, Oike Y (2013). The calcineurin-NFAT-angiopoietin-2 signaling axis in lung endothelium is critical for the establishment of lung metastases. Cell Rep.

[63] Hiratsuka S, Ishibashi S, Tomita T, Watanabe A, Akashi-Takamura S, Murakami M (2013). Primary tumours modulate innate immune signalling to create pre-metastatic vascular hyperpermeability foci. Nat Commun.

[64] Lee E, Fertig EJ, Jin K, Sukumar S, Pandey NB, Popel AS (2014). Breast cancer cells condition lymphatic endothelial cells within pre-metastatic niches to promote metastasis. Nat Commun.

[65] Olmeda D, Cerezo-Wallis D, Riveiro-Falkenbach E, Pennacchi PC, Contreras-Alcalde M, Ibarz N (2017). Whole-body imaging of lymphovascular niches identifies pre-metastatic roles of midkine. Nature.

[66] Chu GC, Zhau HE, Wang R, Rogatko A, Feng X, Zayzafoon M (2014). RANK- and c-Met-mediated signal network promotes prostate cancer metastatic colonization. Endocr Relat Cancer.

[67] Wu JB, Yin L, Shi C, Li Q, Duan P, Huang JM (2017). MAOA-dependent activation of Shh-IL6-RANKL signaling network promotes prostate cancer metastasis by engaging tumor-stromal cell interactions. Cancer Cell.

[68] Sethi N, Dai X, Winter CG, Kang Y (2011). Tumor-derived JAGGED1 promotes osteolytic bone metastasis of breast cancer by engaging notch signaling in bone cells. Cancer Cell.

[69] Lee JW, Stone ML, Porrett PM, Thomas SK, Komar CA, Li JH (2019). Hepatocytes direct the formation of a pro-metastatic niche in the liver. Nature.

[70] Chen XW, Yu TJ, Zhang J, Li Y, Chen HL, Yang GF (2017). CYP4A in tumor-associated macrophages promotes pre-metastatic niche formation and metastasis. Oncogene.

[71] Hiratsuka S, Watanabe A, Sakurai Y, Akashi-Takamura S, Ishibashi S, Miyake K (2008). The S100A8-serum amyloid A3-TLR4 paracrine cascade establishes a pre-metastatic phase. Nat Cell Biol.

[72] Semenza GL (2012). Hypoxia-inducible factors in physiology and medicine. Cell.

[73] Wong CC, Gilkes DM, Zhang H, Chen J, Wei H, Chaturvedi P (2011). Hypoxia-inducible factor 1 is a master regulator of breast cancer metastatic niche formation. Proc Natl Acad Sci U S A.

[74] Erler JT, Bennewith KL, Cox TR, Lang G, Bird D, Koong A (2009). Hypoxia-induced lysyl oxidase is a critical mediator of bone marrow cell recruitment to form the premetastatic niche. Cancer Cell.

[75] Cox TR, Rumney RMH, Schoof EM, Perryman L, Høye AM, Agrawal A (2015). The hypoxic cancer secretome induces pre-metastatic bone lesions through lysyl oxidase. Nature.

[76] Reynaud C, Ferreras L, Di Mauro P, Kan C, Croset M, Bonnelye E (2017). Lysyl oxidase is a strong determinant of tumor cell colonization in bone. Cancer Res.

[77] Wong CC, Tse AP, Huang YP, Zhu YT, Chiu DK, Lai RK (2014). Lysyl oxidase-like 2 is critical to tumor microenvironment and metastatic niche formation in hepatocellular carcinoma. Hepatology.

[78] Chafe SC, Lou Y, Sceneay J, Vallejo M, Hamilton MJ, McDonald PC (2015). Carbonic anhydrase IX promotes myeloid-derived suppressor cell mobilization and establishment of a metastatic niche by stimulating G-CSF production. Cancer Res.

[79] Wang T, Gilkes DM, Takano N, Xiang L, Luo W, Bishop CJ (2014). Hypoxia-inducible factors and RAB22A mediate formation of microvesicles that stimulate breast cancer invasion and metastasis. Proc Natl Acad Sci U S A.

[80] Deep G, Jain A, Kumar A, Agarwal C, Kim S, Leevy WM (2020). Exosomes secreted by prostate cancer cells under hypoxia promote matrix metalloproteinases activity at pre-metastatic niches. Mol Carcinog.

[81] Doglioni G, Parik S, Fendt SM (2019). Interactions in the (pre)metastatic niche support metastasis formation. Front Oncol.

[82] Jiménez-Sánchez A, Memon D, Pourpe S, Veeraraghavan H, Li Y, Vargas HA (2017). Heterogeneous tumor-immune microenvironments among differentially growing metastases in an ovarian cancer patient. Cell.

[83] Kaplan RN, Rafii S, Lyden D (2006). Preparing the “soil”: the premetastatic niche. Cancer Res.

[84] Kitamura T, Qian BZ, Pollard JW (2015). Immune cell promotion of metastasis. Nat Rev Immunol.

[85] Jablonska J, Lang S, Sionov RV, Granot Z (2017). The regulation of pre-metastatic niche formation by neutrophils. Oncotarget.

[86] Shiga K, Hara M, Nagasaki T, Sato T, Takahashi H, Takeyama H (2015). Cancer-associated fibroblasts: their characteristics and their roles in tumor growth. Cancers (Basel).

[87] Chen X, Song E (2019). Turning foes to friends: targeting cancer-associated fibroblasts. Nat Rev Drug Discov.

[88] Monteran L, Erez N (2019). The dark side of fibroblasts: cancer-associated fibroblasts as mediators of immunosuppression in the tumor microenvironment. Front Immunol.

[89] Liu T, Han C, Wang S, Fang P, Ma Z, Xu L (2019). Cancer-associated fibroblasts: an emerging target of anti-cancer immunotherapy. J Hematol Oncol.

[90] Nurmik M, Ullmann P, Rodriguez F, Haan S, Letellier E (2020). In search of definitions: cancer-associated fibroblasts and their markers. Int J Cancer.

[91] Bergers G, Song S (2005). The role of pericytes in blood-vessel formation and maintenance. Neuro-Oncology.

[92] Yamazaki T, Mukouyama YS (2018). Tissue specific origin, development, and pathological perspectives of pericytes. Front Cardiovasc Med.

[93] Ribeiro AL, Okamoto OK (2015). Combined effects of pericytes in the tumor microenvironment. Stem Cells Int.

[94] Guimarães-Camboa N, Cattaneo P, Sun Y, Moore-Morris T, Gu Y, Dalton ND (2017). Pericytes of multiple organs do not behave as mesenchymal stem cells in vivo. Cell Stem Cell.

[95] Birbrair A (2018). Pericyte biology: development, homeostasis, and disease. Adv Exp Med Biol.

[96] Paiva AE, Lousado L, Guerra DAP, Azevedo PO, Sena IFG, Andreotti JP (2018). Pericytes in the premetastatic niche. Cancer Res.

[97] Dias Moura Prazeres PH, Sena IFG, Borges IDT, de Azevedo PO, Andreotti JP, de Paiva AE (2017). Pericytes are heterogeneous in their origin within the same tissue. Dev Biol.

[98] Psaltis PJ, Simari RD (2015). Vascular wall progenitor cells in health and disease. Circ Res.

[99] Mangialardi G, Cordaro A, Madeddu P (2016). The bone marrow pericyte: an orchestrator of vascular niche. Regen Med.

[100] Yamazaki T, Nalbandian A, Uchida Y, Li W, Arnold TD, Kubota Y (2017). Tissue myeloid progenitors differentiate into pericytes through TGF-β signaling in developing skin vasculature. Cell Rep.

[101] Dimmeler S, Zeiher AM (2000). Akt takes center stage in angiogenesis signaling. Circ Res.

[102] Winkler IG, Barbier V, Nowlan B, Jacobsen RN, Forristal CE, Patton JT (2012). Vascular niche E-selectin regulates hematopoietic stem cell dormancy, self renewal and chemoresistance. Nat Med.

[103] Ullah I, Subbarao RB, Rho GJ. Human mesenchymal stem cells - current trends and future prospective. Biosci Rep. 2015;35(2). 10.1042/bsr20150025. 10.1042/BSR20150025 PMC4413017 25797907

[104] Caplan AI (1991). Mesenchymal stem cells. J Orthop Res.

[105] Graham N, Qian BZ. Mesenchymal stromal cells: emerging roles in bone metastasis. Int J Mol Sci. 2018;19(4). 10.3390/ijms19041121. 10.3390/ijms19041121 PMC5979535 29642534

[106] Caplan AI (2008). All MSCs are pericytes?. Cell Stem Cell.

[107] Caplan AI (2017). New MSC: MSCs as pericytes are sentinels and gatekeepers. J Orthop Res.

[108] Rossi MI, Bonfim DC. Mesenchymal stromal/stem cells: historical perspective and ongoing challenges. Braz J Vet Med. 2020;42(1). 10.29374/2527-2179.bjvm112020.

[109] Torsvik A, Bjerkvig R (2013). Mesenchymal stem cell signaling in cancer progression. Cancer Treat Rev.

[110] Karnoub AE, Dash AB, Vo AP, Sullivan A, Brooks MW, Bell GW (2007). Mesenchymal stem cells within tumour stroma promote breast cancer metastasis. Nature.

[111] Mi Z, Bhattacharya SD, Kim VM, Guo H, Talbot LJ, Kuo PC (2011). Osteopontin promotes CCL5-mesenchymal stromal cell-mediated breast cancer metastasis. Carcinogenesis.

[112] Ren G, Esposito M, Kang Y (2015). Bone metastasis and the metastatic niche. J Mol Med (Berl).

[113] Zhang W, Bado I, Wang H, Lo HC, Zhang XH (2019). Bone metastasis: find your niche and fit in. Trends Cancer.

[114] Celià-Terrassa T, Kang Y (2016). Distinctive properties of metastasis-initiating cells. Genes Dev.

[115] Baccelli I, Trumpp A (2012). The evolving concept of cancer and metastasis stem cells. J Cell Biol.

[116] Oskarsson T, Batlle E, Massagué J (2014). Metastatic stem cells: sources, niches, and vital pathways. Cell Stem Cell.

[117] Liu S, Ginestier C, Ou SJ, Clouthier SG, Patel SH, Monville F (2011). Breast cancer stem cells are regulated by mesenchymal stem cells through cytokine networks. Cancer Res.

[118] Plaks V, Kong N, Werb Z (2015). The cancer stem cell niche: how essential is the niche in regulating stemness of tumor cells?. Cell Stem Cell.

[119] Frantz C, Stewart KM, Weaver VM (2010). The extracellular matrix at a glance. J Cell Sci.

[120] Bonnans C, Chou J, Werb Z (2014). Remodelling the extracellular matrix in development and disease. Nat Rev Mol Cell Biol.

[121] Gattazzo F, Urciuolo A, Bonaldo P (2014). Extracellular matrix: a dynamic microenvironment for stem cell niche. Biochim Biophys Acta.

[122] Novoseletskaya ES, Grigorieva OA, Efimenko AY, Kalinina NI (2019). Extracellular matrix in the regulation of stem cell differentiation. Biochemistry (Mosc).

[123] Nusse R, Fuerer C, Ching W, Harnish K, Logan C, Zeng A (2008). Wnt signaling and stem cell control. Cold Spring Harb Symp Quant Biol.

[124] Kai F, Drain AP, Weaver VM (2019). The extracellular matrix modulates the metastatic journey. Dev Cell.

[125] Hynes RO (2009). The extracellular matrix: not just pretty fibrils. Science.

[126] Lu P, Weaver VM, Werb Z (2012). The extracellular matrix: a dynamic niche in cancer progression. J Cell Biol.

[127] Høye AM, Erler JT (2016). Structural ECM components in the premetastatic and metastatic niche. Am J Physiol Cell Physiol.

[128] Paolillo M, Schinelli S. Extracellular matrix alterations in metastatic processes. Int J Mol Sci. 2019;20(19). 10.3390/ijms20194947. 10.3390/ijms20194947 PMC6802000 31591367

[129] Eble JA, Niland S (2019). The extracellular matrix in tumor progression and metastasis. Clin Exp Metastasis.

[130] Egeblad M, Rasch MG, Weaver VM (2010). Dynamic interplay between the collagen scaffold and tumor evolution. Curr Opin Cell Biol.

[131] Gordon MK, Hahn RA (2010). Collagens. Cell Tissue Res.

[132] DeClerck YA (2012). Desmoplasia: a response or a niche?. Cancer Discov.

[133] Xu S, Xu H, Wang W, Li S, Li H, Li T (2019). The role of collagen in cancer: from bench to bedside. J Transl Med.

[134] Lin TC, Yang CH, Cheng LH, Chang WT, Lin YR, Cheng HC. Fibronectin in cancer: friend or foe. Cells. 2019;9(1). 10.3390/cells9010027. 10.3390/cells9010027 PMC7016990 31861892

[135] Libring S, Shinde A, Chanda MK, Nuru M, George H, Saleh AM, et al. The dynamic relationship of breast cancer cells and fibroblasts in fibronectin accumulation at primary and metastatic tumor sites. Cancers (Basel). 2020;12(5). 10.3390/cancers12051270. 10.3390/cancers12051270 PMC7281295 32429591

[136] Wang JP, Hielscher A (2017). Fibronectin: how its aberrant expression in tumors may improve therapeutic targeting. J Cancer.

[137] Fogelgren B, Polgár N, Szauter KM, Ujfaludi Z, Laczkó R, Fong KS (2005). Cellular fibronectin binds to lysyl oxidase with high affinity and is critical for its proteolytic activation. J Biol Chem.

[138] Astachov L, Vago R, Aviv M, Nevo Z (2011). Hyaluronan and mesenchymal stem cells: from germ layer to cartilage and bone. Front Biosci (Landmark Ed).

[139] Zöller M (2015). CD44, hyaluronan, the hematopoietic stem cell, and leukemia-initiating cells. Front Immunol.

[140] Chen C, Zhao S, Karnad A, Freeman JW (2018). The biology and role of CD44 in cancer progression: therapeutic implications. J Hematol Oncol.

[141] Yan Y, Zuo X, Wei D (2015). Concise review: emerging role of CD44 in cancer stem cells: a promising biomarker and therapeutic target. Stem Cells Transl Med.

[142] Williams K, Motiani K, Giridhar PV, Kasper S (2013). CD44 integrates signaling in normal stem cell, cancer stem cell and (pre)metastatic niches. Exp Biol Med (Maywood).

[143] McFarlane S, Coulter JA, Tibbits P, O'Grady A, McFarlane C, Montgomery N (2015). CD44 increases the efficiency of distant metastasis of breast cancer. Oncotarget.

[144] Avigdor A, Goichberg P, Shivtiel S, Dar A, Peled A, Samira S (2004). CD44 and hyaluronic acid cooperate with SDF-1 in the trafficking of human CD34+ stem/progenitor cells to bone marrow. Blood.

[145] Wortzel I, Dror S, Kenific CM, Lyden D (2019). Exosome-mediated metastasis: communication from a distance. Dev Cell.

[146] Carvalho R, Paredes J, Ribeiro AS. Impact of breast cancer cells´ secretome on the brain metastatic niche remodeling. Semin Cancer Biol. 2020;60:294–301. 10.1016/j.semcancer.2019.10.011. 10.1016/j.semcancer.2019.10.011 31711993

[147] Feng W, Dean DC, Hornicek FJ, Shi H, Duan Z (2019). Exosomes promote pre-metastatic niche formation in ovarian cancer. Mol Cancer.

[148] Sceneay J, Smyth MJ, Möller A (2013). The pre-metastatic niche: finding common ground. Cancer Metastasis Rev.

[149] Aguado BA, Bushnell GG, Rao SS, Jeruss JS, Shea LD. Engineering the pre-metastatic niche. Nat Biomed Eng. 2017;1. 10.1038/s41551-017-0077. 10.1038/s41551-017-0077 PMC5628747 28989814

[150] Peinado H, Zhang H, Matei IR, Costa-Silva B, Hoshino A, Rodrigues G (2017). Pre-metastatic niches: organ-specific homes for metastases. Nat Rev Cancer.

[151] Chin AR, Wang SE (2016). Cancer tills the premetastatic field: mechanistic basis and clinical implications. Clin Cancer Res.

[152] Huang Y, Song N, Ding Y, Yuan S, Li X, Cai H (2009). Pulmonary vascular destabilization in the premetastatic phase facilitates lung metastasis. Cancer Res.

[153] Yan HH, Pickup M, Pang Y, Gorska AE, Li Z, Chytil A (2010). Gr-1+CD11b+ myeloid cells tip the balance of immune protection to tumor promotion in the premetastatic lung. Cancer Res.

[154] Qi F, He T, Jia L, Song N, Guo L, Ma X (2015). The miR-30 family inhibits pulmonary vascular hyperpermeability in the premetastatic phase by direct targeting of Skp2. Clin Cancer Res.

[155] Balsat C, Blacher S, Herfs M, Van de Velde M, Signolle N, Sauthier P (2017). A specific immune and lymphatic profile characterizes the pre-metastatic state of the sentinel lymph node in patients with early cervical cancer. Oncoimmunology.

[156] Sun B, Zhou Y, Fang Y, Li Z, Gu X, Xiang J (2019). Colorectal cancer exosomes induce lymphatic network remodeling in lymph nodes. Int J Cancer.

[157] Long Y, Lu Z, Xu S, Li M, Wang X, Zhang Z (2020). Self-delivery micellar nanoparticles prevent premetastatic niche formation by interfering with the early recruitment and vascular destruction of granulocytic myeloid-derived suppressor cells. Nano Lett.

[158] Jackstadt R, van Hooff SR, Leach JD, Cortes-Lavaud X, Lohuis JO, Ridgway RA (2019). Epithelial NOTCH signaling rewires the tumor microenvironment of colorectal cancer to drive poor-prognosis subtypes and metastasis. Cancer Cell.

[159] Sun C, Mezzadra R, Schumacher TN (2018). Regulation and function of the PD-L1 checkpoint. Immunity.

[160] Chen G, Huang AC, Zhang W, Zhang G, Wu M, Xu W (2018). Exosomal PD-L1 contributes to immunosuppression and is associated with anti-PD-1 response. Nature.

[161] Lukanidin E, Sleeman JP (2012). Building the niche: the role of the S100 proteins in metastatic growth. Semin Cancer Biol.

[162] Bresnick AR, Weber DJ, Zimmer DB (2015). S100 proteins in cancer. Nat Rev Cancer.

[163] Tomita T, Sakurai Y, Ishibashi S, Maru Y (2011). Imbalance of Clara cell-mediated homeostatic inflammation is involved in lung metastasis. Oncogene.

[164] Deguchi A, Tomita T, Ohto U, Takemura K, Kitao A, Akashi-Takamura S (2016). Eritoran inhibits S100A8-mediated TLR4/MD-2 activation and tumor growth by changing the immune microenvironment. Oncogene.

[165] Padilla L, Dakhel S, Adan J, Masa M, Martinez JM, Roque L (2017). S100A7: from mechanism to cancer therapy. Oncogene.

[166] Hansen MT, Forst B, Cremers N, Quagliata L, Ambartsumian N, Grum-Schwensen B (2015). A link between inflammation and metastasis: serum amyloid A1 and A3 induce metastasis, and are targets of metastasis-inducing S100A4. Oncogene.

[167] Shu S, Yang Y, Allen CL, Maguire O, Minderman H, Sen A (2018). Metabolic reprogramming of stromal fibroblasts by melanoma exosome microRNA favours a pre-metastatic microenvironment. Sci Rep.

[168] Patel S, Fu S, Mastio J, Dominguez GA, Purohit A, Kossenkov A (2018). Unique pattern of neutrophil migration and function during tumor progression. Nat Immunol.

[169] Michelet X, Dyck L, Hogan A, Loftus RM, Duquette D, Wei K (2018). Metabolic reprogramming of natural killer cells in obesity limits antitumor responses. Nat Immunol.

[170] Loo JM, Scherl A, Nguyen A, Man FY, Weinberg E, Zeng Z (2015). Extracellular metabolic energetics can promote cancer progression. Cell.

[171] Bu P, Chen KY, Xiang K, Johnson C, Crown SB, Rakhilin N (2018). Aldolase B-mediated fructose metabolism drives metabolic reprogramming of colon cancer liver metastasis. Cell Metab.

[172] Wu Z, Wei D, Gao W, Xu Y, Hu Z, Ma Z (2015). TPO-induced metabolic reprogramming drives liver metastasis of colorectal cancer CD110+ tumor-initiating cells. Cell Stem Cell.

[173] Hausser J, Alon U (2020). Tumour heterogeneity and the evolutionary trade-offs of cancer. Nat Rev Cancer.

[174] Granot Z, Henke E, Comen EA, King TA, Norton L, Benezra R (2011). Tumor entrained neutrophils inhibit seeding in the premetastatic lung. Cancer Cell.

[175] Wculek SK, Malanchi I (2015). Neutrophils support lung colonization of metastasis-initiating breast cancer cells. Nature.

[176] Headley MB, Bins A, Nip A, Roberts EW, Looney MR, Gerard A (2016). Visualization of immediate immune responses to pioneer metastatic cells in the lung. Nature.

[177] López-Lago MA, Posner S, Thodima VJ, Molina AM, Motzer RJ, Chaganti RS (2013). Neutrophil chemokines secreted by tumor cells mount a lung antimetastatic response during renal cell carcinoma progression. Oncogene.

[178] Mouchemore KA, Anderson RL, Hamilton JA (2018). Neutrophils, G-CSF and their contribution to breast cancer metastasis. FEBS J.

[179] Schuldner M, Dörsam B, Shatnyeva O, Reiners KS, Kubarenko A, Hansen HP (2019). Exosome-dependent immune surveillance at the metastatic niche requires BAG6 and CBP/p300-dependent acetylation of p53. Theranostics.

[180] Dupuy F, Tabariès S, Andrzejewski S, Dong Z, Blagih J, Annis MG (2015). PDK1-dependent metabolic reprogramming dictates metastatic potential in breast cancer. Cell Metab.

[181] Rachman-Tzemah C, Zaffryar-Eilot S, Grossman M, Ribero D, Timaner M, Mäki JM (2017). Blocking surgically induced lysyl oxidase activity reduces the risk of lung metastases. Cell Rep.

[182] Sosa MS, Bragado P, Aguirre-Ghiso JA (2014). Mechanisms of disseminated cancer cell dormancy: an awakening field. Nat Rev Cancer.

[183] Endo H, Inoue M (2019). Dormancy in cancer. Cancer Sci.

[184] Ghajar CM, Peinado H, Mori H, Matei IR, Evason KJ, Brazier H (2013). The perivascular niche regulates breast tumour dormancy. Nat Cell Biol.

[185] Yu-Lee LY, Yu G, Lee YC, Lin SC, Pan J, Pan T (2018). Osteoblast-secreted factors mediate dormancy of metastatic prostate cancer in the bone via activation of the TGFβRIII-p38MAPK-pS249/T252RB pathway. Cancer Res.

[186] Pommier A, Anaparthy N, Memos N, Kelley ZL, Gouronnec A, Yan R, et al. Unresolved endoplasmic reticulum stress engenders immune-resistant, latent pancreatic cancer metastases. Science. 2018;360(6394). 10.1126/science.aao4908. 10.1126/science.aao4908 PMC6547380 29773669

[187] Eyles J, Puaux AL, Wang X, Toh B, Prakash C, Hong M (2010). Tumor cells disseminate early, but immunosurveillance limits metastatic outgrowth, in a mouse model of melanoma. J Clin Invest.

[188] Liang H, Deng L, Chmura S, Burnette B, Liadis N, Darga T (2013). Radiation-induced equilibrium is a balance between tumor cell proliferation and T cell-mediated killing. J Immunol.

[189] Romero-Moreno R, Curtis KJ, Coughlin TR, Miranda-Vergara MC, Dutta S, Natarajan A (2019). The CXCL5/CXCR2 axis is sufficient to promote breast cancer colonization during bone metastasis. Nat Commun.

[190] Albrengues J, Shields MA, Ng D, Park CG, Ambrico A, Poindexter ME, et al. Neutrophil extracellular traps produced during inflammation awaken dormant cancer cells in mice. Science. 2018;361(6409). 10.1126/science.aao4227. 10.1126/science.aao4227 PMC6777850 30262472

[191] Yuzhalin AE, Gordon-Weeks AN, Tognoli ML, Jones K, Markelc B, Konietzny R (2018). Colorectal cancer liver metastatic growth depends on PAD4-driven citrullination of the extracellular matrix. Nat Commun.

[192] Carlson P, Dasgupta A, Grzelak CA, Kim J, Barrett A, Coleman IM (2019). Targeting the perivascular niche sensitizes disseminated tumour cells to chemotherapy. Nat Cell Biol.

[193] Er EE, Valiente M, Ganesh K, Zou Y, Agrawal S, Hu J (2018). Pericyte-like spreading by disseminated cancer cells activates YAP and MRTF for metastatic colonization. Nat Cell Biol.

[194] Valiente M, Obenauf AC, Jin X, Chen Q, Zhang XH, Lee DJ (2014). Serpins promote cancer cell survival and vascular co-option in brain metastasis. Cell.

[195] Gao D, Joshi N, Choi H, Ryu S, Hahn M, Catena R (2012). Myeloid progenitor cells in the premetastatic lung promote metastases by inducing mesenchymal to epithelial transition. Cancer Res.

[196] Aguirre-Ghiso JA, Estrada Y, Liu D, Ossowski L (2003). ERK(MAPK) activity as a determinant of tumor growth and dormancy; regulation by p38(SAPK). Cancer Res.

[197] Aguirre-Ghiso JA, Liu D, Mignatti A, Kovalski K, Ossowski L (2001). Urokinase receptor and fibronectin regulate the ERK(MAPK) to p38(MAPK) activity ratios that determine carcinoma cell proliferation or dormancy in vivo. Mol Biol Cell.

[198] Bragado P, Estrada Y, Parikh F, Krause S, Capobianco C, Farina HG (2013). TGF-β2 dictates disseminated tumour cell fate in target organs through TGF-β-RIII and p38α/β signalling. Nat Cell Biol.

[199] Sosa MS, Parikh F, Maia AG, Estrada Y, Bosch A, Bragado P (2015). NR2F1 controls tumour cell dormancy via SOX9- and RARβ-driven quiescence programmes. Nat Commun.

[200] Gawrzak S, Rinaldi L, Gregorio S, Arenas EJ, Salvador F, Urosevic J (2018). MSK1 regulates luminal cell differentiation and metastatic dormancy in ER(+) breast cancer. Nat Cell Biol.

[201] Liau BB, Sievers C, Donohue LK, Gillespie SM, Flavahan WA, Miller TE (2017). Adaptive chromatin remodeling drives glioblastoma stem cell plasticity and drug tolerance. Cell Stem Cell.

[202] Roesch A, Vultur A, Bogeski I, Wang H, Zimmermann KM, Speicher D (2013). Overcoming intrinsic multidrug resistance in melanoma by blocking the mitochondrial respiratory chain of slow-cycling JARID1B(high) cells. Cancer Cell.

[203] Dalvi MP, Wang L, Zhong R, Kollipara RK, Park H, Bayo J (2017). Taxane-platin-resistant lung cancers co-develop hypersensitivity to JumonjiC demethylase inhibitors. Cell Rep.

[204] Vinogradova M, Gehling VS, Gustafson A, Arora S, Tindell CA, Wilson C (2016). An inhibitor of KDM5 demethylases reduces survival of drug-tolerant cancer cells. Nat Chem Biol.

[205] Risom T, Langer EM, Chapman MP, Rantala J, Fields AJ, Boniface C (2018). Differentiation-state plasticity is a targetable resistance mechanism in basal-like breast cancer. Nat Commun.

[206] Guler GD, Tindell CA, Pitti R, Wilson C, Nichols K, KaiWai Cheung T (2017). Repression of stress-induced LINE-1 expression protects cancer cell subpopulations from lethal drug exposure. Cancer Cell.

[207] Wang L, Leite de Oliveira R, Huijberts S, Bosdriesz E, Pencheva N, Brunen D (2018). An acquired vulnerability of drug-resistant melanoma with therapeutic potential. Cell.

[208] Heinemann B, Nielsen JM, Hudlebusch HR, Lees MJ, Larsen DV, Boesen T (2014). Inhibition of demethylases by GSK-J1/J4. Nature.

[209] Yang WS, SriRamaratnam R, Welsch ME, Shimada K, Skouta R, Viswanathan VS (2014). Regulation of ferroptotic cancer cell death by GPX4. Cell.

[210] Hangauer MJ, Viswanathan VS, Ryan MJ, Bole D, Eaton JK, Matov A (2017). Drug-tolerant persister cancer cells are vulnerable to GPX4 inhibition. Nature.

[211] Luo M, Brooks M, Wicha MS (2018). Asparagine and glutamine: co-conspirators fueling metastasis. Cell Metab.

[212] Pavlova NN, Hui S, Ghergurovich JM, Fan J, Intlekofer AM, White RM (2018). As extracellular glutamine levels decline, asparagine becomes an essential amino acid. Cell Metab.

[213] Qian BZ, Zhang H, Li J, He T, Yeo EJ, Soong DY (2015). FLT1 signaling in metastasis-associated macrophages activates an inflammatory signature that promotes breast cancer metastasis. J Exp Med.

[214] Freire Valls A, Knipper K, Giannakouri E, Sarachaga V, Hinterkopf S, Wuehrl M (2019). VEGFR1(+) metastasis-associated macrophages contribute to metastatic angiogenesis and influence colorectal cancer patient outcome. Clin Cancer Res.

[215] Chen Q, Zhang XH, Massagué J (2011). Macrophage binding to receptor VCAM-1 transmits survival signals in breast cancer cells that invade the lungs. Cancer Cell.

[216] Etzerodt A, Moulin M, Doktor TK, Delfini M, Mossadegh-Keller N, Bajenoff M, et al. Tissue-resident macrophages in omentum promote metastatic spread of ovarian cancer. J Exp Med. 2020;217(4). 10.1084/jem.20191869. 10.1084/jem.20191869 PMC7144521 31951251

[217] Gordon-Weeks AN, Lim SY, Yuzhalin AE, Jones K, Markelc B, Kim KJ (2017). Neutrophils promote hepatic metastasis growth through fibroblast growth factor 2-dependent angiogenesis in mice. Hepatology.

[218] Tabariès S, Ouellet V, Hsu BE, Annis MG, Rose AA, Meunier L (2015). Granulocytic immune infiltrates are essential for the efficient formation of breast cancer liver metastases. Breast Cancer Res.

[219] Ombrato L, Nolan E, Kurelac I, Mavousian A, Bridgeman VL, Heinze I (2019). Metastatic-niche labelling reveals parenchymal cells with stem features. Nature.

[220] Del Pozo Martin Y, Park D, Ramachandran A, Ombrato L, Calvo F, Chakravarty P (2015). Mesenchymal cancer cell-stroma crosstalk promotes niche activation, epithelial reversion, and metastatic colonization. Cell Rep.

[221] Schwartz H, Blacher E, Amer M, Livneh N, Abramovitz L, Klein A (2016). Incipient melanoma brain metastases instigate astrogliosis and neuroinflammation. Cancer Res.

[222] Nielsen SR, Quaranta V, Linford A, Emeagi P, Rainer C, Santos A (2016). Macrophage-secreted granulin supports pancreatic cancer metastasis by inducing liver fibrosis. Nat Cell Biol.

[223] Elia I, Rossi M, Stegen S, Broekaert D, Doglioni G, van Gorsel M (2019). Breast cancer cells rely on environmental pyruvate to shape the metastatic niche. Nature.

[224] Lemma S, Di Pompo G, Porporato PE, Sboarina M, Russell S, Gillies RJ (2017). MDA-MB-231 breast cancer cells fuel osteoclast metabolism and activity: a new rationale for the pathogenesis of osteolytic bone metastases. Biochim Biophys Acta Mol basis Dis.

[225] Gao Y, Bado I, Wang H, Zhang W, Rosen JM, Zhang XH (2019). Metastasis organotropism: redefining the congenial soil. Dev Cell.

[226] Fornetti J, Welm AL, Stewart SA (2018). Understanding the bone in cancer metastasis. J Bone Miner Res.

[227] Suva LJ, Washam C, Nicholas RW, Griffin RJ (2011). Bone metastasis: mechanisms and therapeutic opportunities. Nat Rev Endocrinol.

[228] Schneider JG, Amend SR, Weilbaecher KN (2011). Integrins and bone metastasis: integrating tumor cell and stromal cell interactions. Bone.

[229] Obenauf AC, Massagué J (2015). Surviving at a distance: organ-specific metastasis. Trends Cancer.

[230] Monteiro AC, Leal AC, Gonçalves-Silva T, Mercadante AC, Kestelman F, Chaves SB (2013). T cells induce pre-metastatic osteolytic disease and help bone metastases establishment in a mouse model of metastatic breast cancer. PLoS One.

[231] Conley-LaComb MK, Semaan L, Singareddy R, Li Y, Heath EI, Kim S (2016). Pharmacological targeting of CXCL12/CXCR4 signaling in prostate cancer bone metastasis. Mol Cancer.

[232] Price TT, Burness ML, Sivan A, Warner MJ, Cheng R, Lee CH (2016). Dormant breast cancer micrometastases reside in specific bone marrow niches that regulate their transit to and from bone. Sci Transl Med.

[233] Wang H, Yu C, Gao X, Welte T, Muscarella AM, Tian L (2015). The osteogenic niche promotes early-stage bone colonization of disseminated breast cancer cells. Cancer Cell.

[234] Lu X, Mu E, Wei Y, Riethdorf S, Yang Q, Yuan M (2011). VCAM-1 promotes osteolytic expansion of indolent bone micrometastasis of breast cancer by engaging α4β1-positive osteoclast progenitors. Cancer Cell.

[235] Taichman RS, Patel LR, Bedenis R, Wang J, Weidner S, Schumann T (2013). GAS6 receptor status is associated with dormancy and bone metastatic tumor formation. PLoS One.

[236] Brodt P (2016). Role of the microenvironment in liver metastasis: from pre- to prometastatic niches. Clin Cancer Res.

[237] Barbazán J, Alonso-Alconada L, Elkhatib N, Geraldo S, Gurchenkov V, Glentis A (2017). Liver metastasis is facilitated by the adherence of circulating tumor cells to vascular fibronectin deposits. Cancer Res.

[238] Achrol AS, Rennert RC, Anders C, Soffietti R, Ahluwalia MS, Nayak L (2019). Brain metastases. Nat Rev Dis Primers.

[239] Arvanitis CD, Ferraro GB, Jain RK (2020). The blood-brain barrier and blood-tumour barrier in brain tumours and metastases. Nat Rev Cancer.

[240] Chen Q, Boire A, Jin X, Valiente M, Er EE, Lopez-Soto A (2016). Carcinoma-astrocyte gap junctions promote brain metastasis by cGAMP transfer. Nature.

[241] Xing F, Liu Y, Sharma S, Wu K, Chan MD, Lo HW (2016). Activation of the c-Met pathway mobilizes an inflammatory network in the brain microenvironment to promote brain metastasis of breast cancer. Cancer Res.

[242] Sevenich L, Bowman RL, Mason SD, Quail DF, Rapaport F, Elie BT (2014). Analysis of tumour- and stroma-supplied proteolytic networks reveals a brain-metastasis-promoting role for cathepsin S. Nat Cell Biol.

[243] Hebert JD, Myers SA, Naba A, Abbruzzese G, Lamar JM, Carr SA (2020). Proteomic profiling of the ECM of xenograft breast cancer metastases in different organs reveals distinct metastatic niches. Cancer Res.

[244] Klotz R, Thomas A, Teng T, Han SM, Iriondo O, Li L (2020). Circulating tumor cells exhibit metastatic tropism and reveal brain metastasis drivers. Cancer Discov.

[245] Murgai M, Ju W, Eason M, Kline J, Beury DW, Kaczanowska S (2017). KLF4-dependent perivascular cell plasticity mediates pre-metastatic niche formation and metastasis. Nat Med.

[246] El Rayes T, Catena R, Lee S, Stawowczyk M, Joshi N, Fischbach C (2015). Lung inflammation promotes metastasis through neutrophil protease-mediated degradation of Tsp-1. Proc Natl Acad Sci U S A.

[247] Salvador F, Martin A, López-Menéndez C, Moreno-Bueno G, Santos V, Vázquez-Naharro A (2017). Lysyl oxidase-like protein LOXL2 promotes lung metastasis of breast cancer. Cancer Res.

[248] Reichert M, Bakir B, Moreira L, Pitarresi JR, Feldmann K, Simon L (2018). Regulation of epithelial plasticity determines metastatic organotropism in pancreatic cancer. Dev Cell.

[249] Gao W, Chen L, Ma Z, Du Z, Zhao Z, Hu Z (2013). Isolation and phenotypic characterization of colorectal cancer stem cells with organ-specific metastatic potential. Gastroenterology.

[250] Stupp R, Hegi ME, Gorlia T, Erridge SC, Perry J, Hong YK (2014). Cilengitide combined with standard treatment for patients with newly diagnosed glioblastoma with methylated MGMT promoter (CENTRIC EORTC 26071-22072 study): a multicentre, randomised, open-label, phase 3 trial. Lancet Oncol.

[251] Masuda T, Noda M, Kogawa T, Kitagawa D, Hayashi N, Jomori T (2020). Phase I dose-escalation trial to repurpose propagermanium, an oral CCL2 inhibitor, in patients with breast cancer. Cancer Sci.

[252] Juergens RA, Wrangle J, Vendetti FP, Murphy SC, Zhao M, Coleman B (2011). Combination epigenetic therapy has efficacy in patients with refractory advanced non-small cell lung cancer. Cancer Discov.

[253] Connolly RM, Li H, Jankowitz RC, Zhang Z, Rudek MA, Jeter SC (2017). Combination epigenetic therapy in advanced breast cancer with 5-azacitidine and entinostat: a phase II national cancer institute/stand up to cancer study. Clin Cancer Res.

[254] Azad NS, El-Khoueiry A, Yin J, Oberg AL, Flynn P, Adkins D (2017). Combination epigenetic therapy in metastatic colorectal cancer (mCRC) with subcutaneous 5-azacitidine and entinostat: a phase 2 consortium/stand up 2 cancer study. Oncotarget.

[255] Coleman R, Finkelstein DM, Barrios C, Martin M, Iwata H, Hegg R (2020). Adjuvant denosumab in early breast cancer (D-CARE): an international, multicentre, randomised, controlled, phase 3 trial. Lancet Oncol.

[256] Gnant M, Pfeiler G, Steger GG, Egle D, Greil R, Fitzal F (2019). Adjuvant denosumab in postmenopausal patients with hormone receptor-positive breast cancer (ABCSG-18): disease-free survival results from a randomised, double-blind, placebo-controlled, phase 3 trial. Lancet Oncol.

[257] Smith MR, Saad F, Coleman R, Shore N, Fizazi K, Tombal B (2012). Denosumab and bone-metastasis-free survival in men with castration-resistant prostate cancer: results of a phase 3, randomised, placebo-controlled trial. Lancet.

[258] Angevin E, Tabernero J, Elez E, Cohen SJ, Bahleda R, van Laethem JL (2014). A phase I/II, multiple-dose, dose-escalation study of siltuximab, an anti-interleukin-6 monoclonal antibody, in patients with advanced solid tumors. Clin Cancer Res.

[259] Hecht JR, Benson AB, Vyushkov D, Yang Y, Bendell J, Verma U (2017). A phase II, randomized, double-blind, placebo-controlled study of simtuzumab in combination with FOLFIRI for the second-line treatment of metastatic KRAS mutant colorectal adenocarcinoma. Oncologist.

[260] Benson AB, Wainberg ZA, Hecht JR, Vyushkov D, Dong H, Bendell J (2017). A phase II randomized, double-blind, placebo-controlled study of simtuzumab or placebo in combination with gemcitabine for the first-line treatment of pancreatic adenocarcinoma. Oncologist.

[261] Shah MA, Yanez Ruiz EP, Bodoky G, Starodub A, Cunningham D, Yip D (2019). A phase III, randomized, double-blind, placebo-controlled study to evaluate the efficacy and safety of andecaliximab combined with mFOLFOX6 as first-line treatment in patients with advanced gastric or gastroesophageal junction adenocarcinoma (GAMMA-1). J Clin Oncol.

[262] Halama N, Zoernig I, Berthel A, Kahlert C, Klupp F, Suarez-Carmona M (2016). Tumoral immune cell exploitation in colorectal cancer metastases can be targeted effectively by anti-CCR5 therapy in cancer patients. Cancer Cell.

[263] Melisi D, Garcia-Carbonero R, Macarulla T, Pezet D, Deplanque G, Fuchs M (2018). Galunisertib plus gemcitabine vs. gemcitabine for first-line treatment of patients with unresectable pancreatic cancer. Br J Cancer.

[264] Nishida-Aoki N, Tominaga N, Takeshita F, Sonoda H, Yoshioka Y, Ochiya T (2017). Disruption of circulating extracellular vesicles as a novel therapeutic strategy against cancer metastasis. Mol Ther.

[265] Ortiz A, Gui J, Zahedi F, Yu P, Cho C, Bhattacharya S (2019). An interferon-driven oxysterol-based defense against tumor-derived extracellular vesicles. Cancer Cell.

[266] Ye H, Wang K, Lu Q, Zhao J, Wang M, Kan Q (2020). Nanosponges of circulating tumor-derived exosomes for breast cancer metastasis inhibition. Biomaterials.

[267] Zhao L, Gu C, Gan Y, Shao L, Chen H, Zhu H (2020). Exosome-mediated siRNA delivery to suppress postoperative breast cancer metastasis. J Control Release.

[268] Bonapace L, Coissieux MM, Wyckoff J, Mertz KD, Varga Z, Junt T (2014). Cessation of CCL2 inhibition accelerates breast cancer metastasis by promoting angiogenesis. Nature.

[269] Yumimoto K, Akiyoshi S, Ueo H, Sagara Y, Onoyama I, Ueo H (2015). F-box protein FBXW7 inhibits cancer metastasis in a non-cell-autonomous manner. J Clin Invest.

[270] Lu Z, Zou J, Li S, Topper MJ, Tao Y, Zhang H (2020). Epigenetic therapy inhibits metastases by disrupting premetastatic niches. Nature.

[271] Low V, Blenis J, Gomes AP (2020). Targeting the premetastatic niche: epigenetic therapies in the spotlight. Signal Transduct Target Ther.

[272] Steele CW, Karim SA, Leach JDG, Bailey P, Upstill-Goddard R, Rishi L (2016). CXCR2 inhibition profoundly suppresses metastases and augments immunotherapy in pancreatic ductal adenocarcinoma. Cancer Cell.

[273] Chen IX, Chauhan VP, Posada J, Ng MR, Wu MW, Adstamongkonkul P (2019). Blocking CXCR4 alleviates desmoplasia, increases T-lymphocyte infiltration, and improves immunotherapy in metastatic breast cancer. Proc Natl Acad Sci U S A.

[274] Lacey DL, Boyle WJ, Simonet WS, Kostenuik PJ, Dougall WC, Sullivan JK (2012). Bench to bedside: elucidation of the OPG-RANK-RANKL pathway and the development of denosumab. Nat Rev Drug Discov.

[275] Vanharanta S, Massagué J (2013). Origins of metastatic traits. Cancer Cell.

[276] Lee W, Ko SY, Mohamed MS, Kenny HA, Lengyel E, Naora H (2019). Neutrophils facilitate ovarian cancer premetastatic niche formation in the omentum. J Exp Med.

[277] Yang L, Liu Q, Zhang X, Liu X, Zhou B, Chen J, et al. DNA of neutrophil extracellular traps promotes cancer metastasis via CCDC25. Nature. 2020. 10.1038/s41586-020-2394-6. 10.1038/s41586-020-2394-6 32528174

[278] Park J, Wysocki RW, Amoozgar Z, Maiorino L, Fein MR, Jorns J (2016). Cancer cells induce metastasis-supporting neutrophil extracellular DNA traps. Sci Transl Med.

[279] Teijeira Á, Garasa S, Gato M, Alfaro C, Migueliz I, Cirella A, et al. CXCR1 and CXCR2 chemokine receptor agonists produced by tumors induce neutrophil extracellular traps that interfere with immune cytotoxicity. Immunity. 2020. 10.1016/j.immuni.2020.03.001. 10.1016/j.immuni.2020.03.001 32289253

[280] Wu CF, Andzinski L, Kasnitz N, Kröger A, Klawonn F, Lienenklaus S (2015). The lack of type I interferon induces neutrophil-mediated pre-metastatic niche formation in the mouse lung. Int J Cancer.

[281] Wang Y, Chu Y, Ren X, Xiang H, Xi Y, Ma X (2019). Epidural adipose tissue-derived mesenchymal stem cell activation induced by lung cancer cells promotes malignancy and EMT of lung cancer. Stem Cell Res Ther.

[282] Jing B, Wang T, Sun B, Xu J, Xu D, Liao Y (2020). IL6/STAT3 signaling orchestrates premetastatic niche formation and immunosuppressive traits in lung. Cancer Res.

[283] Milagre CS, Gopinathan G, Everitt G, Thompson RG, Kulbe H, Zhong H (2015). Adaptive upregulation of EGFR limits attenuation of tumor growth by neutralizing IL6 antibodies, with implications for combined therapy in ovarian cancer. Cancer Res.

[284] Zheng H, Bae Y, Kasimir-Bauer S, Tang R, Chen J, Ren G (2017). Therapeutic antibody targeting tumor- and osteoblastic niche-derived Jagged1 sensitizes bone metastasis to chemotherapy. Cancer Cell.

[285] Wong CC, Zhang H, Gilkes DM, Chen J, Wei H, Chaturvedi P (2012). Inhibitors of hypoxia-inducible factor 1 block breast cancer metastatic niche formation and lung metastasis. J Mol Med (Berl).

[286] Cox TR, Bird D, Baker AM, Barker HE, Ho MW, Lang G (2013). LOX-mediated collagen crosslinking is responsible for fibrosis-enhanced metastasis. Cancer Res.

[287] Nilsson M, Adamo H, Bergh A, Halin Bergström S (2016). Inhibition of lysyl oxidase and lysyl oxidase-like enzymes has tumour-promoting and tumour-suppressing roles in experimental prostate cancer. Sci Rep.

[288] Wu S, Zheng Q, Xing X, Dong Y, Wang Y, You Y (2018). Matrix stiffness-upregulated LOXL2 promotes fibronectin production, MMP9 and CXCL12 expression and BMDCs recruitment to assist pre-metastatic niche formation. J Exp Clin Cancer Res.

[289] Kim H, Chung H, Kim J, Choi DH, Shin Y, Kang YG (2019). Macrophages-triggered sequential remodeling of endothelium-interstitial matrix to form pre-metastatic niche in microfluidic tumor microenvironment. Adv Sci (Weinh).

[290] McCloskey CW, Cook DP, Kelly BS, Azzi F, Allen CH, Forsyth A (2020). Metformin abrogates age-associated ovarian fibrosis. Clin Cancer Res.

[291] Kang SA, Hasan N, Mann AP, Zheng W, Zhao L, Morris L (2015). Blocking the adhesion cascade at the premetastatic niche for prevention of breast cancer metastasis. Mol Ther.

[292] Morita Y, Kamal M, Kang SA, Zhang R, Lokesh GL, Thiviyanathan V (2016). E-selectin targeting PEGylated-thioaptamer prevents breast cancer metastases. Mol Ther Nucleic Acids.

[293] Neman J, Termini J, Wilczynski S, Vaidehi N, Choy C, Kowolik CM (2014). Human breast cancer metastases to the brain display GABAergic properties in the neural niche. Proc Natl Acad Sci U S A.

[294] de la Fuente A, Alonso-Alconada L, Costa C, Cueva J, Garcia-Caballero T, Lopez-Lopez R, et al. M-trap: exosome-based capture of tumor cells as a new technology in peritoneal metastasis. J Natl Cancer Inst. 2015;107(9). 10.1093/jnci/djv184. 10.1093/jnci/djv184 PMC4836824 26150590

[295] Srivastava K, Hu J, Korn C, Savant S, Teichert M, Kapel SS (2014). Postsurgical adjuvant tumor therapy by combining anti-angiopoietin-2 and metronomic chemotherapy limits metastatic growth. Cancer Cell.

[296] Roblek M, Calin M, Schlesinger M, Stan D, Zeisig R, Simionescu M (2015). Targeted delivery of CCR2 antagonist to activated pulmonary endothelium prevents metastasis. J Control Release.

[297] Ghouse SM, Vadrevu SK, Manne S, Reese B, Patel J, Patel B (2020). Therapeutic targeting of vasculature in the premetastatic and metastatic niches reduces lung metastasis. J Immunol.

[298] He B, Johansson-Percival A, Backhouse J, Li J, Lee GYF, Hamzah J (2020). Remodeling of metastatic vasculature reduces lung colonization and sensitizes overt metastases to immunotherapy. Cell Rep.

[299] Lucotti S, Cerutti C, Soyer M, Gil-Bernabé AM, Gomes AL, Allen PD (2019). Aspirin blocks formation of metastatic intravascular niches by inhibiting platelet-derived COX-1/thromboxane A2. J Clin Invest.

[300] Liu Y, Kosaka A, Ikeura M, Kohanbash G, Fellows-Mayle W, Snyder LA (2013). Premetastatic soil and prevention of breast cancer brain metastasis. Neuro-Oncology.

[301] Gu Y, Liu Y, Fu L, Zhai L, Zhu J, Han Y (2019). Tumor-educated B cells selectively promote breast cancer lymph node metastasis by HSPA4-targeting IgG. Nat Med.

[302] Olkhanud PB, Damdinsuren B, Bodogai M, Gress RE, Sen R, Wejksza K (2011). Tumor-evoked regulatory B cells promote breast cancer metastasis by converting resting CD4^+^ T cells to T-regulatory cells. Cancer Res.

[303] Bodogai M, Moritoh K, Lee-Chang C, Hollander CM, Sherman-Baust CA, Wersto RP (2015). Immunosuppressive and prometastatic functions of myeloid-derived suppressive cells rely upon education from tumor-associated B cells. Cancer Res.

[304] Bodogai M, Lee Chang C, Wejksza K, Lai J, Merino M, Wersto RP (2013). Anti-CD20 antibody promotes cancer escape via enrichment of tumor-evoked regulatory B cells expressing low levels of CD20 and CD137L. Cancer Res.

[305] Lee-Chang C, Bodogai M, Martin-Montalvo A, Wejksza K, Sanghvi M, Moaddel R (2013). Inhibition of breast cancer metastasis by resveratrol-mediated inactivation of tumor-evoked regulatory B cells. J Immunol.

[306] Herbertz S, Sawyer JS, Stauber AJ, Gueorguieva I, Driscoll KE, Estrem ST (2015). Clinical development of galunisertib (LY2157299 monohydrate), a small molecule inhibitor of transforming growth factor-beta signaling pathway. Drug Des Devel Ther.

[307] Yan HH, Jiang J, Pang Y, Achyut BR, Lizardo M, Liang X (2015). CCL9 induced by TGFβ signaling in myeloid cells enhances tumor cell survival in the premetastatic organ. Cancer Res.

[308] Clever D, Roychoudhuri R, Constantinides MG, Askenase MH, Sukumar M, Klebanoff CA (2016). Oxygen sensing by T Cells establishes an immunologically tolerant metastatic niche. Cell.

[309] Coffelt SB, Kersten K, Doornebal CW, Weiden J, Vrijland K, Hau CS (2015). IL-17-producing γδ T cells and neutrophils conspire to promote breast cancer metastasis. Nature.

[310] Cheng R, Billet S, Liu C, Haldar S, Choudhury D, Tripathi M (2020). Periodontal inflammation recruits distant metastatic breast cancer cells by increasing myeloid-derived suppressor cells. Oncogene.

[311] Shi H, Zhang J, Han X, Li H, Xie M, Sun Y (2017). Recruited monocytic myeloid-derived suppressor cells promote the arrest of tumor cells in the premetastatic niche through an IL-1β-mediated increase in E-selectin expression. Int J Cancer.

[312] Kaplanov I, Carmi Y, Kornetsky R, Shemesh A, Shurin GV, Shurin MR (2019). Blocking IL-1β reverses the immunosuppression in mouse breast cancer and synergizes with anti-PD-1 for tumor abrogation. Proc Natl Acad Sci U S A.

